# Functional characterization of a lytic polysaccharide monooxygenase from *Schizophyllum commune* that degrades non-crystalline substrates

**DOI:** 10.1038/s41598-023-44278-1

**Published:** 2023-10-13

**Authors:** Heidi Østby, Idd A. Christensen, Karen Hennum, Anikó Várnai, Edith Buchinger, Siri Grandal, Gaston Courtade, Olav A. Hegnar, Finn L. Aachmann, Vincent G. H. Eijsink

**Affiliations:** 1 Faculty of Chemistry, Biotechnology, and Food Science, Norwegian University of Life Sciences (NMBU), P.O. Box 5003, 1432 Ås, Norway; 2 Norwegian Biopolymer Laboratory (NOBIPOL), Department of Biotechnology and Food Science, NTNU Norwegian University of Science and Technology, Sem Sælands Vei 6/8, 7491 Trondheim, Norway

**Keywords:** Carbohydrates, Enzymes

## Abstract

Lytic polysaccharide monooxygenases (LPMOs) are mono-copper enzymes that use O_2_ or H_2_O_2_ to oxidatively cleave glycosidic bonds. LPMOs are prevalent in nature, and the functional variation among these enzymes is a topic of great interest. We present the functional characterization of one of the 22 putative AA9-type LPMOs from the fungus *Schizophyllum commune*, *Sc*LPMO9A. The enzyme, expressed in *Escherichia coli*, showed C4-oxidative cleavage of amorphous cellulose and soluble cello-oligosaccharides. Activity on xyloglucan, mixed-linkage β-glucan, and glucomannan was also observed, and product profiles differed compared to the well-studied C4-oxidizing *Nc*LPMO9C from *Neurospora crassa*. While *Nc*LPMO9C is also active on more crystalline forms of cellulose, *Sc*LPMO9A is not. Differences between the two enzymes were also revealed by nuclear magnetic resonance (NMR) titration studies showing that, in contrast to *Nc*LPMO9C, *Sc*LPMO9A has higher affinity for linear substrates compared to branched substrates. Studies of H_2_O_2_-fueled degradation of amorphous cellulose showed that *Sc*LPMO9A catalyzes a fast and specific peroxygenase reaction that is at least two orders of magnitude faster than the apparent monooxygenase reaction. Together, these results show that *Sc*LPMO9A is an efficient LPMO with a broad substrate range, which, rather than acting on cellulose, has evolved to act on amorphous and soluble glucans.

## Introduction

Since their discovery in 2010, lytic polysaccharide monooxygenases (LPMOs) have been the focus of much research with the aim of better understanding their unique properties and harnessing their oxidative power^1–4^. LPMOs are commonly associated with the conversion of recalcitrant insoluble polysaccharides such as cellulose and chitin. However, several LPMOs belonging to the well-studied AA9 family of fungal cellulose-active LPMOs are active on hemicelluloses and cello-oligomers, while a few LPMOs acting primarily on pectin and starch have been described^5^. LPMOs are abundant in nature, with some fungal genomes coding for dozens of LPMOs, and the true roles and substrates of many of these enzymes likely remain undiscovered^6–8^.

LPMOs are copper-dependent redox enzymes that use an oxidative mechanism (monooxygenase- or peroxygenase-type activity) to catalyze the scission of polysaccharide glycosidic bonds^1,9–11^. The active site of LPMOs contains a histidine brace consisting of two conserved histidine residues that coordinate the copper atom^9,12,13^. LPMO catalysis requires reduction of the copper, which may be achieved by small-molecule reductants such as ascorbic acid, gallic acid, or cysteine, enzymatic electron donors such as cellobiose dehydrogenase, or redox-active compounds in the substrate itself, such as lignin^1,9,14–17^. In the presence of a relevant substrate, reduced LPMOs can utilize either molecular O_2_ or H_2_O_2_ as a co-substrate to catalyze the hydroxylation of a carbon in the scissile glycosidic bond (C1 or C4 in cellulose), leading to spontaneous bond cleavage^9,10,18^. Once reduced, a single LPMO molecule acting as a peroxygenase can catalyze multiple turnovers^10,19^. Non-substrate bound reduced LPMOs in solution can react with O_2_ to produce H_2_O_2_^20,21^, or with H_2_O_2_, generating reactive oxygen species that may lead to damage and autocatalytic inactivation^10^. While the significance of the monooxygenase vs. peroxygenase reaction is still under debate, it is worth noting that LPMO reactions with H_2_O_2_ are several orders of magnitude faster than those driven by O_2_^4,19,22–26^.

LPMOs currently populate eight of the 17 families in the auxiliary activities (AA) class of the carbohydrate-active enzymes (CAZy) database (http://www.cazy.org/;^27^). This class encompasses oxidases, peroxidases, and oxidoreductases in addition to LPMOs^28^. Most characterized fungal LPMOs can be found in the AA9 family, which at the time of writing contains more than 60 functionally characterized LPMOs with activities on insoluble and soluble cellulosic and hemicellulosic substrates. The N-terminal histidine of AA9 LPMOs, which is part of the copper-binding histidine brace, carries a methylation^12^, which helps to protect the enzymes from oxidative damage^29^. Of note, non-methylated variants of these LPMOs have been produced in the yeast *Pichia pastoris* and are active. Well-studied examples of AA9 LPMOs include *Nc*LPMO9C from *Neurospora crassa*, *Ls*LPMO9A from *Lentinus similis*, and *Cv*LPMO9A from *Collariella virescens*^26,30–34^, which stand out due to their proven ability to act on soluble substrates, including cello-oligomers, and hemicelluloses such as glucomannan and xyloglucan.

The ability of LPMOs to boost the action of canonical glycoside hydrolases makes them interesting candidates for use in the valorization of recalcitrant polysaccharides in lignocellulosic biomass^2,35,36^. Indeed, modern cellulase cocktails used in lignocellulosic biorefineries contain LPMOs, and their contribution to cellulose saccharification efficiency is evident^3,37–39^. So far, LPMO action in bioprocessing has exclusively been focused on oxidative degradation of cellulose, whereas the potential impact of hemicellulolytic LPMO activities, if present in commercial cocktails, has not been addressed. The continued elucidation of novel LPMOs acting on various lignocellulosic polysaccharides may provide novel tools for biomass processing and may help understand the biological reasons for the large LPMO multiplicity observed in some fungal species.

Basidiomycete wood-decaying filamentous fungi are a rich source of enzymes for the depolymerization of complex plant matter, including LPMOs. The genome of one such basidiomycete fungus, *Schizophyllum commune*, was first sequenced in 2010, and showed, in addition to genes coding for an extensive array of glycoside hydrolases active on cellulose, xylan, and pectin, the presence of genes encoding 22 putative AA9s^40^. A comparative study of four fungi including *S. commune* by Zhu and colleagues indicated that the *S. commune* secretome had significantly higher cellulase and xylanase activities than other white- and brown-rot fungi tested during solid-state fermentation of Jerusalem artichoke stalk. Proteomic analysis of the *S. commune* secretome revealed the presence of a wide range of cellulolytic and hemicellulolytic enzymes, and eight AA9s, including *Sc*LPMO9A. In addition, a crude secretome-based enzyme cocktail from *S. commune* outperformed a commercial enzyme blend from *Trichoderma longibrachiatum* in saccharifications of multiple lignocellulosic substrates, both in conversion of glucan and xylan^41^. A comparative study of *S. commune* and the closely related *Auriculariopsis ampla* showed that the gene encoding *Sc*LPMO9A and the orthologous gene in *A. ampla* are among the most highly upregulated AA9 genes in vegetative mycelium growing on poplar wood^42^.

As an AA9 candidate for in-depth characterization, *Sc*LPMO9A is of particular interest, as it is produced by *S. commune* under different conditions and when grown on different substrates, hinting at a crucial role of this enzyme during growth and nutrient acquisition. In this study we have cloned, produced, and purified *Sc*LPMO9A, and performed an in-depth functional characterization of this enzyme. The properties of this single-domain AA9 LPMO, active on soluble substrates, are compared to the properties of the well-studied *Nc*LPMO9C. We show that *Sc*LPMO9A is active on and interacts with a range of soluble and amorphous substrates, whereas its activity on crystalline cellulose substrates is limited, suggesting that this enzyme’s natural role is not in saccharification of recalcitrant cellulose. Additionally, we show that *Sc*LPMO9A is a rapid consumer of H_2_O_2_, both in reactions with soluble cellopentaose and in the oxidative depolymerization of amorphous cellulose.

## Materials and methods

### Sequence and structure analysis

A multiple sequence alignment (MSA) was created using T-Coffee Expresso (http://tcoffee.crg.cat/apps/tcoffee/index.html;^43^) by aligning the sequence of *Sc*LPMO9A with 46 other characterized AA9s, using only the AA9 domains and removing signal peptides. The MSA was edited in AliView^44^, and the resulting MSA was used for phylogenetic analysis using the ProtTest 3.4 software package, by calculating likelihood scores using all included substitution matrices, all improvements, and four categories for rate variation, empirical amino acid frequencies, and a fixed BIONJ JTT tree for base likelihood calculations^45^. A consensus tree was built with all 120 likelihood scores using the Akaike information criterion (AIC). The resulting consensus tree was edited using the iTol v6 online tool (https://itol.embl.de/;^46^). Homology modeling of *Sc*LPMO9A using *Ls*LPMO9A bound to cellohexaose (PDB ID 5ACI; 61.1% sequence identity) as a template structure was performed using PHYRE2^47^, and the resulting model was analyzed in PyMOL (The PyMOL Molecular Graphics System, Version 2.0, Schrödinger, LLC).

### Protein expression and purification

A gene fragment containing the signal peptide pelB (MKYLLPTAAAGLLLLAAQPAMA)^48^ fused with the gene encoding *Sc*LPMO9A (UniProt ID D8Q364; residues 20-247) was codon-optimized for expression in *Escherichia coli* and de novo synthesized by GenScript (Piscataway, NJ, USA). Restriction sites for *Nde*I and *Not*I were included upstream and downstream of the coding area, respectively. The pelB-*Sc*LPMO9A fragment was isolated from the Genscript vector by digestion with *Nde*I and *Not*I, and ligated into the compatible *Nde*I and *Not*I sites of the pJB_SP_*Sm*-vector^49^ (replacing the SP_*Sm* gene fragment), generating pJB_pelB_*Sc*. The pJB_pelB_*Sc* plasmid was transformed into competent *E. coli* T7 express cells (New England Biolabs, Ipswich, MA, USA) using a heat shock protocol. Plasmid DNA was isolated from the cells using the Wizard® Plus SV Minipreps DNA purification system (Promega, Madison, WI, USA), and the plasmid was verified by full vector sequencing.

Expression of non-labeled and ^15^N-isotopically labeled *Sc*LPMO9A was performed as described earlier for other LPMOs^49^. The cell pellet was harvested by centrifugation at 6000 × g and subjected to osmotic shock to prepare a periplasmic extract^50^, which was filtered using a 0.22 µm sterile filter.

Purification of *Sc*LPMO9A was performed by anion-exchange chromatography using an Äkta Purifier system with a 5 mL HiTrap DEAE FF column (GE Healthcare, Uppsala, Sweden), equilibrated with 50 mM Tris–HCl pH 7.5. After loading the sterile-filtered periplasmic extract onto the column, *Sc*LPMO9A was eluted using a 0–500 mM NaCl gradient with 50 mM Tris–HCl pH 7.5 over 90 column volumes. Protein purity was assessed using SDS-PAGE. Fractions containing *Sc*LPMO9A were pooled, buffer exchanged to 50 mM Tris–HCl pH 7.5, and concentrated using a 10-kDa Vivaspin centrifugal tube with PES membrane (Sartorius, Göttingen, Germany) at 10 °C and 6000 × g. For the ^15^N-labeled protein used in NMR studies, an additional purification step using size-exclusion chromatography (SEC) was applied. The fractions containing ^15^N-labeled *Sc*LPMO9A (identified by SDS-PAGE) were pooled and concentrated to < 5 mL using a 10-kDa Vivaspin centrifugal tube as described above. The concentrated sample was used for further purification by SEC using a HiLoad® 16/600 Superdex® G-75 pg column equilibrated with 25 mM Tris–HCl pH 8.0, 250 mM NaCl with a flow rate of 1 mL/min. Fractions containing ^15^N-*Sc*LPMO9A were pooled and buffer exchanged to 25 mM Tris–HCl pH 7.0, 25 mM NaCl, 2.3% glycerol, and concentrated to a final volume of approximately 160 µL as described above. The protein concentration was determined spectrophotometrically at 280 nm using the theoretical molar extinction coefficient (51,005 M^−1^·cm^−1^), determined using the ExPASy ProtParam tool^51^.

Expression and purification of *Nc*LPMO9C were performed as described earlier^25^, and copper saturation of both LPMOs was performed as previously described^52^.

The correct amino acid sequence of purified *Sc*LPMO9A was verified by subjecting the purified LPMO to trypsination, as well as extraction and clean-up of the resulting peptides as previously described^53^. Peptides were subsequently analyzed via liquid chromatography-tandem mass spectrometry using a nano UPLC (nanoElute, Bruker Daltonics GmbH, Bremen, Germany) coupled to a trapped ion mobility spectrometry/quadrupole time-of-flight mass spectrometer (timsTOF Pro, Bruker Daltonics GmbH). Peptide separation was achieved using a PepSep Reprosil C18 reverse-phase (1.5 µm, 100 Å) 25 cm × 75 μm analytical column kept at 50 °C coupled to a ZDV Sprayer (Bruker Daltonics GmbH). Prior to sample loading, the column was equilibrated using a pressure of 800 bar. Peptides were separated using an operational flow of 300 nL/min and a 60 min solvent gradient (0–40 min, from 5 to 25% B; 40–45 min, from 25 to 37% B; 45–50 min, from 37 to 95% B; 50–60 min, constant at 95% B). Solvent A consisted of 0.1% (v/v) formic acid in distilled H_2_O (type I, 18.2 MΩ·cm), while the composition of solvent B was 0.1% (v/v) formic acid in acetonitrile. The timsTOF Pro was run in positive ion data-dependent acquisition PASEF mode, using the control softwares Compass Hystar version 5.1.8.1 and timsControl version 1.1.19, and with an acquisition mass range of 100–1700 *m/z*. The TIMS settings were: 1/K0 start 0.85 V⋅s/cm^2^ and 1/K0 end 1.4 V⋅s/cm^2^, ramp time 100 ms, ramp rate 9.42 Hz, duty cycle 100%. The capillary voltage was set to 1400 V, dry gas to 3.0 L/min, and dry temp to 180 °C. MS/MS settings were as follows: number of PASEF ramps 10, total cycle time 0.53 s, charge range 0–5, scheduling target intensity 20,000, intensity threshold 2500, active exclusion release after 0.4 min, CID collision energy ranging from 27 to 45 eV. The *E. coli* proteome (UniProt ID UP000002032) was used as reference. We obtained 70% sequence coverage and the obtained sequence, inferred from a combination of MS/MS-based sequencing and peptide masses, was identical to that of UniProt ID D8Q364.

### Substrates and chemicals

Cellulosic substrates used in this study included Avicel PH-101 (Sigma-Aldrich, St. Louis, MO, USA), PASC (prepared from Avicel as described in ^54^), cellotetraose, cellopentaose, and cellohexaose (all purchased from Megazyme, Wicklow, Ireland), and sulfite-pulped spruce (batch number DP3319; composition in % w/w dry matter: 87.4% glucan, 2.7% xylan, 5.2% mannan, and 3.3% lignin), kindly provided by Borregaard AS^55,56^. Hemicellulosic substrates used were low-viscosity konjac glucomannan (KGM), xyloglucan from tamarind seed (TXG), medium-viscosity mixed-linkage β-glucan from barley (β(1,3;1,4)-glucan; MLBG), higher DP xyloglucan oligomers (xyloglucan tetradecamer; XG14), birchwood xylan, beechwood xylan, and low-viscosity arabinoxylan from wheat flour. All hemicellulosic substrates were purchased from Megazyme. XG14 (Product number O-XGHDP) is a mixture of xyloglucan oligomers with the sequence XXXGXXXG, where G denotes an unsubstituted glucose monomer and X denotes a glucose monomer with a xylosyl substitution. In addition, up to three of the X units can be further substituted with galactose (denoted as L).

Ascorbic acid (AscA) was used as a reducing agent in all LPMO reactions. Aliquots of a stock solution of 100 mM AscA prepared in TraceSELECT water (Sigma-Aldrich) were prepared and stored at − 20 °C. Aliquots were thawed in the dark immediately prior to use.

### Production and consumption of H_2_O_2_

An adapted version of the Amplex Red assay^20^ was used to quantify H_2_O_2_ production by *Sc*LPMO9A and *Nc*LPMO9C. Reaction mixtures contained 3 µM LPMO, 100 µM Amplex Red (Thermo Fisher Scientific, Waltham, MA, USA), 0.5 U horseradish peroxidase (Sigma-Aldrich), and 50 µM AscA in 50 mM BisTris-HCl pH 6.5, and reactions were initiated by the addition of AscA. The reactions were incubated at 30 °C in a Varioscan LUX plate reader (Thermo Fisher Scientific), and the production of resorufin was measured spectrophotometrically at 563 nm every 22 s over a total time of 6500 s. Control reactions containing 3 µM CuSO_4_ in place of the LPMO were performed in parallel.

An assay adapted from Breslmayr et al.^57^ was used to measure H_2_O_2_ consumption by the LPMOs. Reaction mixtures contained 3 µM LPMO, 1 mM 2,6-dimethoxyphenol (Sigma-Aldrich), and 100 µM H_2_O_2_ in 50 mM BisTris-HCl pH 6.5, and reactions were initiated by addition of the LPMO. Reactions were incubated at 30 °C in a Varioscan LUX plate reader (Thermo Fisher Scientific), and the absorbance at 469 nm was measured every 30 s over a total time of 600 s. Control reactions containing 3 µM CuSO_4_ in place of the LPMO were performed in parallel.

### Determination of the redox potential

The cell potential for the redox couple *Sc*LPMO9A-Cu^2+^/*Sc*LPMO9A-Cu^1+^ was determined as previously described^31,58^. Oxygen-free solutions of 300 µM reduced N,N,N’,N’-tetramethyl-1,4-phenylenediamine (TMPred) (Sigma-Aldrich) (30 µL) and 70 µM Cu^2+^-saturated *Sc*LPMO9A (30 µL) were mixed in UVettes (Eppendorf, Hamburg, Germany) in 20 mM PIPES pH 6.0, and incubated at 28 °C under anaerobic conditions. Absorbance at 610 nm was measured using a NanoPhotometer C40 (Implen GmbH, München, Germany) until the signal became stable (5 min). The extinction coefficient of oxidized TMP (TMPox) (14.0 mM^−1^ cm^−1^^59^) was used to calculate the concentration of TMPox, which is equal to the concentration of *Sc*LPMO9A-Cu^1+^. Finally, the cell potential of the *Sc*LPMO9A-Cu^2+^/*Sc*LPMO9A-Cu^1+^ couple was determined using the previously determined cell potential of TMPox/TMPred (273 mV^60^).

### LPMO reactions with cellulosic and hemicellulosic substates

*Sc*LPMO9A activity was tested with a wide range of cellulosic and hemicellulosic substrates. Reactions with *Nc*LPMO9C were included for comparative purposes. Reactions containing 1 µM *Sc*LPMO9A or *Nc*LPMO9C and individual substrates, or hemicellulosic substrates in combination with PASC, were incubated in 50 mM BisTris-HCl pH 6.5 at 40 °C and 1000 rpm in a Thermomixer (Eppendorf) for 16 h. In reactions with polymeric cellulosic substrates and with hemicellulosic substrates, the substrate concentration was 2 g/L or 4 g/L (with the exception of reactions with sulfite-pulped spruce, which contained 10 g/L substrate). In reactions containing a mixture of PASC and hemicellulosic substrate, the final concentration of both substrates was 2 g/L (total substrate content 4 g/L). In reactions with soluble oligomeric cellulose substrates (cellotetraose, cellopentaose, and cellohexaose), the substrate concentration was either 2 g/L or 1 mM. Reactions were initiated by the addition of 1 mM AscA and stopped by removing insoluble substrates by filtration using a 96-well filter plate (0.45 µm; Merck Millipore, Billerica, MA, USA) operated with a Millipore vacuum manifold system. In the case of soluble substrates, reactions were stopped by boiling for 10 min before filtration. Samples were subsequently stored at – 20 °C prior to analysis by high-performance anion exchange chromatography with pulsed amperometric detection (HPAEC-PAD) and/or matrix-assisted laser desorption/ionization time-of-flight mass spectrometry (MALDI-TOF MS). All reactions were performed in triplicate, and control reactions without addition of AscA, or only containing relevant substrate(s) and 1 mM AscA, were performed in parallel.

### H_2_O_2_-driven activity on PASC and cellopentaose

To assess the impact of H_2_O_2_ on product generation by *Sc*LPMO9A acting on PASC, reactions containing 1 µM LPMO, 2 g/L PASC, 1 mM AscA, and 0, 50, 100, or 250 µM H_2_O_2_ in 50 mM Tris–HCl pH 7.5 were prepared. The reactions were initiated by addition of AscA and incubated at 45 °C and 1000 rpm in a Thermomixer (Eppendorf). H_2_O_2_ was added to the reactions immediately prior to the AscA. Samples were taken at 3, 6, 9, 30, and 60 min, and remaining insoluble substrate was removed by filtration using a 96-well filter plate (0.45 µm; Merck Millipore) operated with a Millipore vacuum manifold system. Samples were subsequently stored at – 20 °C prior to analysis by HPAEC-PAD. All reactions were performed in triplicate, and control reactions without addition of AscA were performed in parallel.

Reactions with cellopentaose contained 1 µM LPMO, 1 mM cellopentaose, 50 µM AscA, and 200 or 400 µM H_2_O_2_ in 50 mM sodium acetate pH 5.0. Immediately following the addition of H_2_O_2_, reactions were initiated by addition of AscA and incubated as described above. Samples were taken at various time points and reactions were quenched by addition of NaOH to a final concentration of 100 mM. Samples were subsequently stored at – 20 °C prior to analysis of generated native products by HPAEC-PAD. All reactions were performed in triplicate, and control reactions without addition of AscA were performed in parallel.

### Synergy with cellulases

Degradation of sulfite-pulped spruce was performed under aerobic conditions in 60 mL screw-cap glass bottles (Wheaton, Millville, NJ, USA) using a working volume of 10 mL. The total enzyme loading was 4 mg protein per g dry matter of substrate, and the substrate content was 10% w/w dry matter. The enzymes added were a 9:1 (based on protein content) mix of Celluclast 1.5 L and Novozym 188, both kindly provided by Novozymes AS (Bagsværd, Denmark), and the protein concentrations of these enzyme preparations were determined using the Bio-Rad protein assay (Bio-Rad Laboratories, Hercules, CA, USA), based on the Bradford method^61^, using bovine serum albumin as reference protein. Reactions containing *Sc*LPMO9A contained 3.6 mg of the Celluclast 1.5 L/Novozym 188 blend and 0.4 mg *Sc*LPMO9A per gram of dry matter (based on protein content). The reactions with sulfite-pulped spruce were initiated by the addition of 1 mM AscA. All reactions were incubated at 50 °C with orbital shaking at 200 rpm in a Minitron Shaker incubator (Infors AG, Bottmingen, Switzerland). Reactions were performed in duplicate, and control reactions without addition of AscA were performed in parallel. Samples of 100 µL were taken at 8, 24, 48, and 72 h, and diluted three times in distilled H2O (type I, 18.2 MΩ·cm) prior to enzyme inactivation by boiling for 15 min before storage at – 20 °C. Prior to product quantification, samples were thawed at 4 °C and filtered using a 96-well filter plate (0.45 µm; Merck Millipore) operated with a Millipore vacuum manifold system. Quantification of glucose and cellobiose released during saccharification was performed by high-performance liquid chromatography using a Dionex Ultimate 3000 system (Dionex, Sunnyvale, CA, USA) equipped with a Shodex RI-101 refractive index detector (Shodex, Tokyo, Japan). A Rezex ROA-organic acid H^+^ (8%) 300 × 7.8 mm analytical column (Phenomenex, Torrance, CA, USA) was used, operated at 65 °C with 5 mM H_2_SO_4_ and an isocratic flow of 0.6 mL/min^38^. Cellobiose levels were below 1 g/L in all samples and are not reported. Glc4gemGlc was quantified by HPAEC-PAD as described below.

### Chromatographic analysis of LPMO-derived products by HPAEC-PAD

Products generated in LPMO reactions were analyzed using high-performance anion exchange chromatography with pulsed amperometric detection (HPAEC-PAD) using a Dionex ICS-5000 system (Thermo Fisher Scientific). The ICS-5000 was equipped with a 3 × 250 mm Dionex CarboPac PA-200 analytical column with a 3 × 50 mm guard column (Thermo Fisher Scientific), and the flow rate was 500 µL/min. Eluents (A: 0.1 M NaOH, B: 0.1 M NaOH containing 1 M NaOAc) were prepared as described previously^62^. All samples were diluted two times in distilled water (type I, 18.2 MΩ·cm) prior to analysis using either a 14-min or a 39-min gradient. The 14-min elution profile used was: 0–5 min, convex upward (Dionex curve 4) from 100% A to 90% A and 10% B; 5–8.5 min, concave upward (Dionex curve 8) from 90% A and 10% B to 100% B; 8.5–8.6 min, linear from 100% B to 100% A; 8.6–14 min, constant at 100% A (reconditioning). The 39-min elution profile has previously been described^63^. Chromeleon version 7.2.9 (Thermo Fisher Scientific) was used for instrument control and analysis. Cellobiose and cellotriose used to prepare standards for quantification of native products generated from cellopentaose by LPMO action were purchased from Megazyme. C4-oxidized standards for quantification of Glc4gemGlc, and for identification of Glc4gemGlc and Glc4gemGlc_2_ in product mixtures generated from cellulosic substrates, were produced in-house as previously described^64^.

### Product analysis by MALDI-TOF MS

LPMO products from selected samples were analyzed by matrix-assisted laser desorption/ionization time-of-flight mass spectrometry (MALDI-TOF MS) using an Ultraflex instrument (Bruker Daltonics GmbH) with a Nitrogen 337 nm laser, as described previously^65^. Sample preparation, data collection, and analysis were performed as previously described^63^.

### NMR

Samples of ^15^N-labeled copper-free *Sc*LPMO9A (100 – 140 µM) were prepared in a Tris–HCl buffer (25 mM Tris–HCl pH 7.0, 25 mM NaCl, 2.3% glycerol) with 10% D_2_O (d-99.9%, Sigma-Aldrich) in 3-mm NMR Essence tubes (Bruker Labscape). NMR spectra were recorded at 25 °C on an 800 MHz Bruker Ascent Advance III HD spectrometer equipped with a 5 mm Z-gradient CP-TCI (H/C/N) cryogenic probe using TopSpin version 3.5 pl7. These analyses were carried out at the NV-NMR center node of the Norwegian NMR platform at NTNU, the Norwegian University of Science and Technology. Two-dimensional ^15^N-HSQC (Heteronuclear Single Quantum Coherence) spectra in combination with three-dimensional HNCO and HNCA spectra were recorded to distinguish backbone amide pairs (^1^H-^15^N) from side chains. The NMR data were processed using Bruker TopSpin version 4.1.0 and analyzed using CARA version 1.9.1.7.

### Titration studies by NMR

Interactions between ^15^N-labeled copper-free *Sc*LPMO9A and cellopentaose, cellohexaose, and XG14 were investigated by recording ^15^N-HSQC spectra for different substrate concentrations and measuring the chemical shift perturbation (CSP) of the signals compared with ^15^N-HSQC spectra recorded in the absence of substrate. Different ligand concentrations had to be used because of differences in the K_d_ values for the different substrates. For cellopentaose, the titration points were 0.05, 0.1, 0.2, 0.4, and 1 mM. For cellohexaose, the titration points were 0.1, 0.3, 0.5, 1.1, and 2.5 mM. For XG14, the titration points were 0.4, 0.8, 1, 1.5, 2, 4, 6, and 8 mM.

XG14 is a mixture of xyloglucan oligomers, mainly comprised of oligomers with the sequence XXXGXXXG, where X denotes a glucose with a xylosyl substitution. Based on this, a molecular weight of 2108 g/mol was used in our calculations. Of note, up to three of the X units can be further galactosylated; this was not taken into account in our calculations.

The CSP was calculated using the equation^66^:

$$\Delta {\updelta }_{{{\text{comb}}}} = \sqrt {\left( {\left( {\Delta {\delta H}} \right)^{2} + \left( {\frac{{\Delta {\delta N}}}{6.5}} \right)^{2} } \right)}$$

where Δδ_comb_ is the absolute change in the chemical shift given in hertz (Hz). ΔδH and ΔδN are the changes in the chemical shift of the amide proton (Hz) and amide nitrogen (Hz), respectively. A significant CSP was determined to be Δδ_comb_ ≥ 0.025 ppm for ^1^H-^15^N signals.

The affinity of copper-free ^15^N-labeled *Sc*LPMO9A towards cellopentaose, cellohexaose, and XG14 was quantified by calculating the dissociation constant (K_d_). The K_d_ was determined by plotting the Δδ_comb_ as a function of the substrate concentration for each titration point and fitting the datapoints to the equation:

$$\Delta {\updelta }_{comb} = \Delta {\updelta }_{\max } \frac{{\left[ P \right] + \left[ L \right] + Kd \pm \sqrt {\left[ P \right] + \left[ L \right] + Kd^{2} - 4\left[ P \right]\left[ L \right]} }}{2\left[ P \right]}$$

where [P] and [L] are the protein and ligand concentrations, respectively, Δδ_comb_ is the absolute change in chemical shift (in ppm), and Δδ_max_ is Δδ_comb_ obtained when the enzyme is substrate-saturated^67^. The K_d_ was estimated using the signals of four ^1^H-^15^N pairs in the titration experiments with cellohexaose and XG14, and of two ^1^H-^15^N pairs for titrations with cellopentaose, to provide a K_d_ range.

## Results and discussion

### Analysis of the structure and sequence of *Sc*LPMO9A

**Figure 1:**
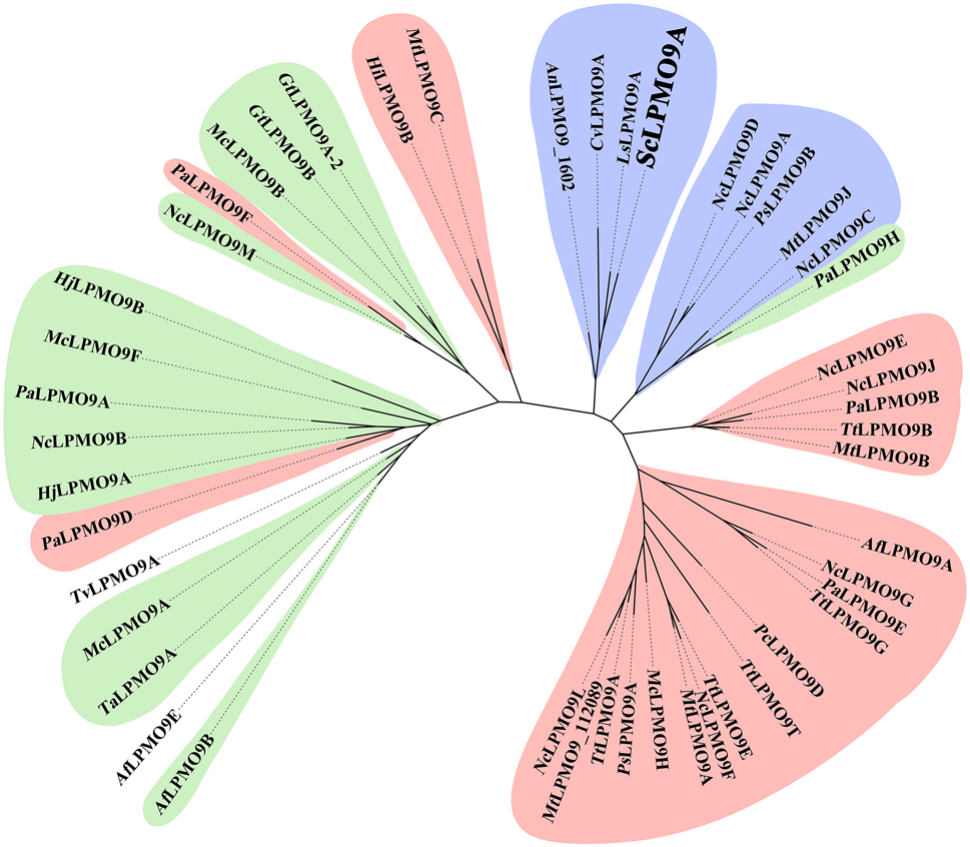
Phylogenetic tree of selected AA9 LPMOs. A multiple-sequence alignment of the catalytic domains of 47 LPMOs, including *Sc*LPMO9A and *Nc*LPMO9C, was performed using T-Coffee Expresso and used to create the phylogenetic tree. The colors in the tree represent LPMO regioselectivity on cellulose (blue: C4-oxidizing; red: C1-oxidizing; green: C1/C4-oxidizing; no color: regioselectivity unknown). All four AA9s grouped in the clade containing *Sc*LPMO9A are active on soluble cello-oligosaccharides.

Phylogenetic analysis of the *Sc*LPMO9A sequence, shown in Fig. 1, indicated that this enzyme clusters with *Ls*LPMO9A and *Cv*LPMO9A, which are C4-oxidizing LPMOs active on soluble cello-oligosaccharides, mixed-linkage β-glucan, glucomannan, and xyloglucan^32,33^. Of note, a comparative functional characterization study of *Ls*LPMO9A and *Cv*LPMO9A by Simmons et al*.* indicated that *Ls*LPMO9A potentially has low activity on birchwood xylan, but this specificity was not detected for *Cv*LPMO9A^33^. The third LPMO clustering with *Sc*LPMO9A (Fig. 1), *An*LPMO9A_1602, is the only one in this cluster with a carbohydrate-binding module (belonging to CAZy family 1, CBM1), and has also been reported to cleave cellohexaose^68^. The well-studied C4-oxidizing CBM1-containing *Nc*LPMO9C from *Neurospora crassa*, shown to be active on cellopentaose, cellohexaose, and to a lesser extent, cellotetraose, in addition to xyloglucan, mixed-linkage β-glucan, and glucomannan^30,65^, appears in the neighboring cluster.

**Figure 2:**
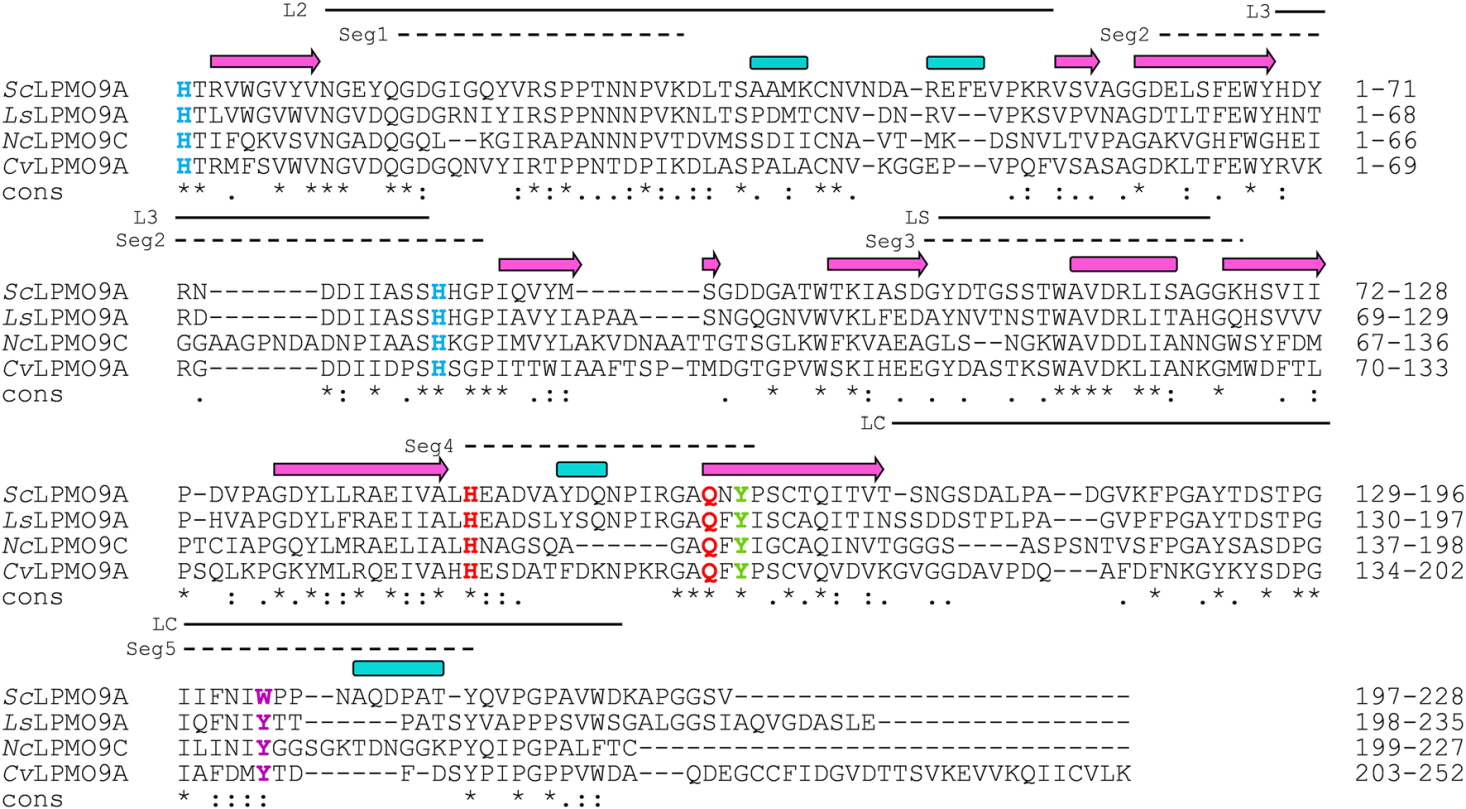
Multiple-sequence alignment (MSA) of the catalytic domains of the C4-oxidizing AA9s *Sc*LPMO9A, *Ls*LPMO9A, *Nc*LPMO9C, and *Cv*LPMO9A. Sequence identities between *Sc*LPMO9A and the other AA9s are as follows: *Ls*LPMO9A 61.1%, *Cv*LPMO9A 46.6%, *Nc*LPMO9C 45.6%. Fully conserved residues are indicated by an asterisk (*). Active site histidines are colored blue, and the conserved tyrosine (Tyr163 in *Sc*LPMO9A) that helps shaping the copper site is colored green. Two highly conserved second sphere residues near the copper site (His146 and Gln161) are colored red, whereas a semi-conserved aromatic residue likely involved in substrate-binding (Trp202; see main text) is colored purple. Pink arrows and blue rectangles above the amino acid sequences indicate predicted secondary structure elements (strands and helices, respectively) in the model of *Sc*LPMO9A made using the structure of *Ls*LPMO9A as a template (Fig. 3). Lines above the sequences represent variable regions in AA9 LPMOs as classified by^88^ (L2, L3, LS, LC) and^89^ (Seg. 1–5).

**Figure 3:**
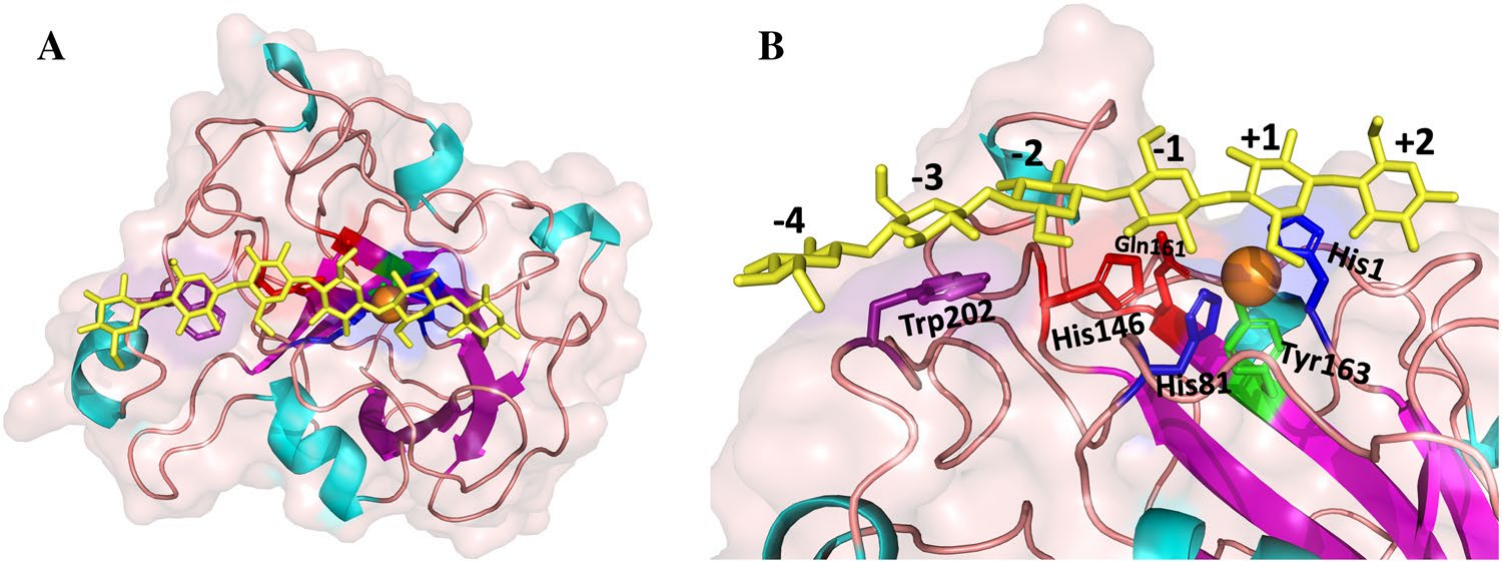
Structural representation of *Sc*LPMO9A seen from the top (A) and a close-up of the substrate binding surface (B). The model was made with PHYRE2^47^ using the structure of *Ls*LPMO9A (5ACI) as a template. The copper ion coordinated in the active site is shown as an orange sphere. Secondary structure elements are shown in light blue (helices), magenta (strands), and light pink (loop regions). The side chains of the active site histidines are colored dark blue, and the side chain of the tyrosine in the proximal axial copper coordination position is colored green. The side chains of His146 and Gln161 are colored red, while the side chain of Trp202 is colored purple. A bound cellohexaose unit, with subsite labelling, is shown in yellow. See main text for more details; additional views comparing the surfaces of *Sc*LPMO9A and *Nc*LPMO9C are provided in the section on the study of binding interactions with NMR below.

A comparison of the sequences of *Sc*LPMO9A, *Ls*LPMO9A, *Cv*LPMO9A, and *Nc*LPMO9C (Fig. 2) shows that residues that make up the histidine brace (His1 and His81), as well as residues in the second coordination sphere of the copper (His146, Gln161, Tyr163), are conserved in *Sc*LPMO9A. Interestingly, *Sc*LPMO9A has a tryptophan (Trp202) at a solvent-exposed position where other AA9 LPMOs, including *Ls*LPMO9A, *Nc*LPMO9C, and *Cv*LPMO9A, tend to have a tyrosine. Previous studies have shown that this exposed aromatic residue interacts with bound oligomeric substrates^32,69^ (Fig. 3). The MSA further shows an alanine residue (Ala78 in *Sc*LPMO9A) shared between *Sc*LPMO9A, *Ls*LPMO9A, and *Nc*LPMO9C, known to be common among C4-oxidizing LPMOs, although this correlation is not absolute (e.g., *Cv*LPMO9A has an Asp in this position^31^). The conserved Ser residue (Ser80 in *Sc*LPMO9A) adjacent to the second histidine of the histidine brace is prevalent in C4-oxidizing LPMOs^70^. In their study of *Cv*LPMO9A and *Ls*LPMO9A, Simmons et al*.* noted that (weak) xylan oxidation was only observed for *Ls*LPMO9A. The authors speculated that this may be due to differences in substrate binding residues of the + 2 subsite (Asn28, His66, and Asn67 in *Ls*LPMO9A, compared to Thr28, Arg67, and Val68 in *Cv*LPMO9A)^33^. *Sc*LPMO9A shares two out of three of these residues with *Ls*LPMO9A (Asn28 and His69), but has an Asp70 in place of the Asn.

A model of *Sc*LPMO9A made using *Ls*LPMO9A bound to cellohexaose as a template, shown in Fig. 3, depicts a shallow groove type surface topology, similar to what has been reported for *Ls*LPMO9A^32^. This shallow groove topology differs somewhat from the characteristically flat binding surfaces of LPMOs known for their activity on crystalline substrates^71^. Docking of a hexameric substrate by superposition with the structure of *Ls*LPMO9A bound to cellohexaose showed that the hexamer fits well in the shallow groove of *Sc*LPMO9A, and that binding interactions seen in *Ls*LPMO9A appear to be preserved in *Sc*LPMO9A. Studies of the interactions between *Sc*LPMO9A and oligosaccharides by NMR titration experiments are described below.

### Production and consumption of H_2_O_2_, and redox potential

In order to verify that *E. coli*-expressed *Sc*LPMO9A was correctly folded and copper-saturated, and to ensure it produced and consumed H_2_O_2_ in a manner expected of AA9 LPMOs, we tested *Sc*LPMO9A in assays adapted from Kittl et al.^20^ and Breslmayr et al.^57^. The former assay couples H_2_O_2_-production by the LPMO (i.e., oxidase activity) to oxidation of Amplex Red by horseradish peroxidase, which can be monitored spectrophotometrically. The latter assay enables spectrophotometric detection of the formation of coerulignone resulting from H_2_O_2_-dependent oxidation of 2,6-dimethoxyphenol by the LPMO. Purified, copper-saturated *Nc*LPMO9C produced in *P. pastoris* was included for comparative purposes. In both assays, *Sc*LPMO9A performed similarly to *Nc*LPMO9C and in accordance with what has previously been reported for AA9 LPMOs, including for *Nc*LPMO9C^8,25^, indicating that *Sc*LPMO9A was properly folded and contained a coordinated copper in its active site.

The redox potential of *Sc*LPMO9A was determined to be 186 ± 10 mV, which is a common, albeit rather low value for AA9 LPMOs. For comparison, using the same method, the redox potential of *Nc*LPMO9C was determined to be 224 ± 3 mV^31^.

### Mapping activity on cellulosic substrates

To begin mapping the substrate specificity of *Sc*LPMO9A, three insoluble cellulosic substrates (Avicel, sulfite-pulped spruce, and PASC) were tested. Cellopentaose was also included given the activity of *Sc*LPMO9A homologs on cellodextrins.

**Figure 4:**
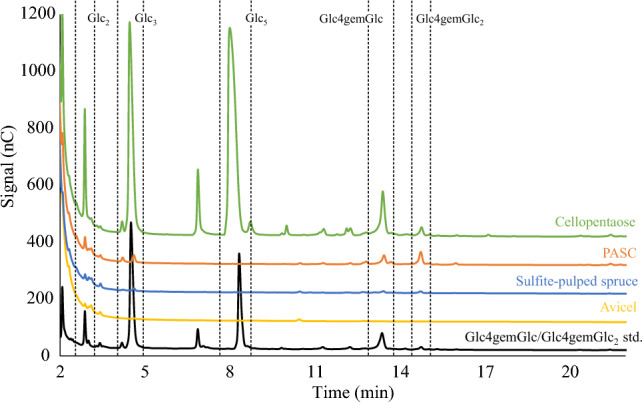
HPAEC-PAD chromatograms of products generated in reactions of *Sc*LPMO9A with cellopentaose (Glc_5_; green), PASC (orange), sulfite-pulped spruce (blue), and Avicel (yellow). Substrate names are provided directly above chromatograms in colors corresponding to the chromatogram. Key products derived from LPMO activity (Glc_2_, Glc_3_, Glc4gemGlc, Glc4gemGlc_2_), and Glc_5_ are indicated within dashed rectangles. The black chromatogram shows a standard containing Glc4gemGlc and Glc4gemGlc_2_ (cellopentaose treated with *Nc*LPMO9C^38^). Note that the C4-oxidized products are unstable and that the products labeled as Glc4gemGlc and Glc4gemGlc_2_ in fact are diagnostic derivatives of these products, while additional products resulting from tautomerization are visible as minor peaks^30,72^. Also note that the peak heights of oxidized and non-oxidized products cannot be compared due to the instability of the former, and due to different response factors. All reactions were performed with 1 µM LPMO, 2 g/L substrate (or 10 g/L for sulfite-pulped spruce), and 1 mM AscA in 50 mM BisTris-HCl pH 6.5, and were incubated at 40 °C and 1000 rpm for 16 h. Control reactions lacking AscA did not show any formation of native or C4-oxidized products. All reactions were carried out in triplicate and gave identical product profiles.

HPAEC-PAD analysis of product formation after 16 h of incubation (Fig. 4) showed that *Sc*LPMO9A, like *Ls*LPMO9A^26,32^ and *Nc*LPMO9C^30^, is a C4-oxidizing cellulose-active LPMO, as evidenced by the reductant-dependent accumulation of signals representing the C4-oxidized products Glc4gemGlc and Glc4gemGlc_2_ in reactions with cellopentaose and PASC. C4-oxidized oligomers are unstable under the conditions used here and are spontaneously converted to various products including those giving the diagnostic peaks for Glc4gemGlc and Glc4gemGlc_2_ and, notably, also including native oligomers lacking the C4-oxidized sugar unit (so, Glc4gemGlc_2_ may be converted to Glc_2_; see also legend to Fig. 4;^72^). No C1-oxidized reaction products were detected for any of the substrates tested. No activity was detected in reactions with the crystalline model substrate Avicel, in contrast to what has been observed for *Nc*LPMO9C, which is active on PASC, Avicel, and cellulose in steam-exploded spruce^30^. The activity of *Sc*LPMO9A on sulfite-pulped spruce, with an expected crystallinity almost as high as Avicel^73^, was also low, suggesting that *Sc*LPMO9A has a preference for amorphous cellulose, as present in PASC. The main C4-oxidized product generated from cellopentaose was the C4-oxidized dimer, and, accordingly, this reaction mixture showed much more cellotriose than cellobiose, where the latter would be the co-product in a reaction leading to the C4-oxidized trimer. This shows that the pentameric substrate preferentially binds from -3 to + 2, similar to what has been observed for *Nc*LPMO9C.

To further investigate the activity on soluble cello-oligomers and examine possible differences between *Sc*LPMO9A and *Nc*LPMO9C, reactions with cellotetraose and cellohexaose were analyzed.

**Figure 5:**
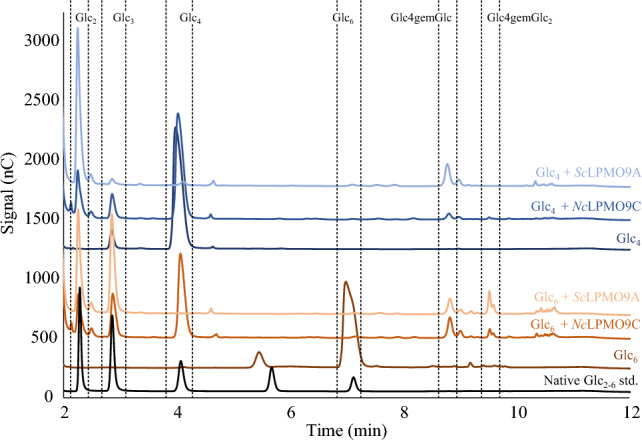
HPAEC-PAD chromatograms for reactions of *Sc*LPMO9A or *Nc*LPMO9C with cellotetraose (Glc_4_) or cellohexaose (Glc_6_). Sample identities are provided directly above chromatograms in colors corresponding to the relevant chromatogram. Key products derived from LPMO activity (Glc_2_, Glc_3_, Glc_4_, Glc4gemGlc, Glc4gemGlc_2_), and Glc_6_ are indicated within dashed rectangles. Reactions contained 1 mM soluble substrate, 1 µM LPMO, and 1 mM AscA in 50 mM BisTris-HCl pH 6.5, and were incubated at 40 °C and 1000 rpm for 16 h. Dark red and dark blue chromatograms show cellohexaose (Glc_6_) and cellotetraose (Glc_4_), respectively, incubated with AscA and without LPMO. A standard consisting of native cello-oligomers from Glc_2–6_ is shown in black. Control reactions lacking AscA did not show any formation of native or C4-oxidized products for either LPMO. All reactions were carried out in triplicate and gave identical product profiles.

Figure 5 shows clear differences between *Sc*LPMO9A and *Nc*LPMO9C. As expected based on previous results^30^, *Nc*LPMO9C showed limited activity on cellotetraose (Glc_4_), generating only minor amounts of Glc4gemGlc and native cellobiose after 16 h of incubation. *Sc*LPMO9A, on the other hand, completely degraded cellotetraose, producing a mixture of cellobiose and Glc4gemGlc. With cellohexaose (Glc_6_), *Nc*LPMO9C generated primarily Glc4gemGlc and cellotetraose, and lesser amounts of cellotriose and Glc4gemGlc_2_, indicating a preference for − 4–+ 2 binding. For *Sc*LPMO9A, productive − 3–+ 3 binding was more prominent, as shown by the relatively large amounts of native trimer and Glc4gemGlc_2_. Whether the native dimer and the oxidized dimer generated by this enzyme with cellohexaose result from − 4–+ 2 or – 2–+ 4 binding cannot be determined based on these experiments, since the tetrameric product may be cleaved by *Sc*LPMO9A. While more work is needed to precisely map and quantify the oligomer binding preferences of *Sc*LPMO9A, it is clear that these differ from those of *Nc*LPMO9C.

### Activity on hemicellulosic substrates

The ability of *Sc*LPMO9A to degrade hemicellulosic substrates was assessed and compared with that of *Nc*LPMO9C. Both LPMOs were tested on konjac glucomannan (KGM), mixed-linkage β-glucan (MLBG), tamarind xyloglucan (TXG), and xyloglucan tetradecamer (XG14), alone or in combination with PASC, as various studies have shown that the presence of cellulose may promote LPMO activity on (presumably cellulose-bound) hemicelluloses^8,63,74,75^. Products from reactions containing LPMO, AscA, and hemicellulosic substrate, hemicellulose + PASC, or PASC were analyzed by HPAEC-PAD and, in some cases, MALDI-TOF MS. Since these are single time point measurements and since LPMOs are prone to inactivation, quantitative interpretation of the results presented below requires great care^76^. However, since a suitable substrate protects LPMOs from inactivation, it is safe to assume that major differences in product levels reflect differences in substrate specificity (a better substrate will give more products and the LPMO is less prone to inactivation).

**Figure 6:**
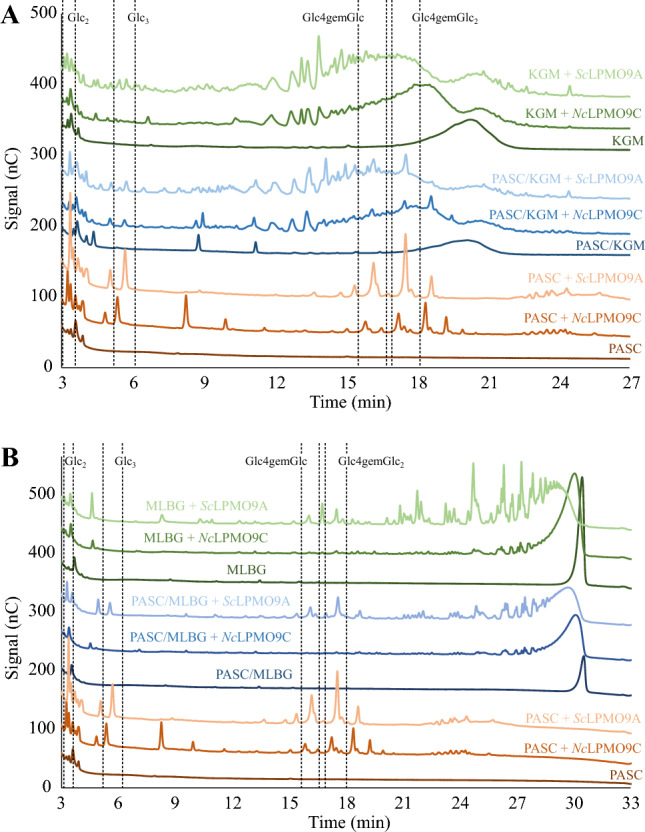
HPAEC-PAD chromatograms for reactions with *Sc*LPMO9A or *Nc*LPMO9C and PASC, konjac glucomannan (KGM), mixed-linkage (β-1,3;1,4) glucan (MLBG), or mixtures of PASC and KGM or MLBG. Panel A shows reactions with PASC and KGM, while Panel B shows reactions with PASC and MLBG. Reaction set-ups are indicated directly above chromatograms in colors corresponding to the relevant chromatogram. Reactions contained 1 µM LPMO, 1 mM AscA, and either 2 g/L KGM or MLBG, or, in the reactions containing hemicellulosic substrate and PASC, 2 g/L of each substrate (4 g/L total substrate concentration). Reactions with PASC alone contained 4 g/L PASC. Reactions lacking LPMO contained the aforementioned concentrations of substrate and 1 mM AscA. Reactions were incubated in 50 mM BisTris-HCl pH 6.5 at 40 °C and 1000 rpm for 16 h. Key products derived from LPMO activity on PASC (Glc2, Glc3, Glc4gemGlc, Glc4gemGlc2) are indicated within dashed rectangles (note that there is a slight shift between the PASC + *Sc*LPMO9A and the PASC + *Nc*LPMO9C chromatograms). Control reactions in the absence of AscA did not show any product formation for either LPMO. All reactions were carried out in triplicate and gave similar product profiles.

Reactions with KGM showed activity of *Sc*LPMO9A and this activity seemed hardly affected by the presence of PASC (Fig. 6A). The product profiles of *Sc*LPMO9A and *Nc*LPMO9C show differences that may indicate differences in substrate-binding preferences and abilities. In particular, *Sc*LPMO9A generates more early-eluting products (5–10 min region). It is also worth noting the substantially higher peak intensities for products generated by *Sc*LPMO9A acting on KGM alone compared to the analogous reaction with *Nc*LPMO9C. The data thus indicate that the two LPMOs have different affinities for glucomannan and/or that they have different cleavage pattern preferences.

Figure 6B shows that *Sc*LPMO9A is clearly more active on MLBG than *Nc*LPMO9C, both in reactions with MLBG alone and in reactions with MLBG and PASC. The activity difference is most pronounced in the reactions with MLBG alone, since the reaction of *Sc*LPMO9A with a mixture of PASC and MLBG yielded less MLBG-derived products than the reaction with MLBG alone. The chromatograms for the reactions with *Sc*LPMO9A show a larger variety of products as compared to *Nc*LPMO9C, although this may partly be a false impression due to the general difference in activity. However, one clear and striking difference stands out: when acting on MLBG alone, in contrast to *Nc*LPMO9C, *Sc*LPMO9A generates a relatively high amount of products eluting in the 15–19 min region, which likely are C4-oxidized glucan fragments such as Glc4gemGlc and Glc4gemGlc_2_. This indicates that *Sc*LPMO9A has a greater ability to convert MLBG to small oligomeric products and is, thus, less inhibited by the β-(1,3) bonds in MLBG.

**Figure 7:**
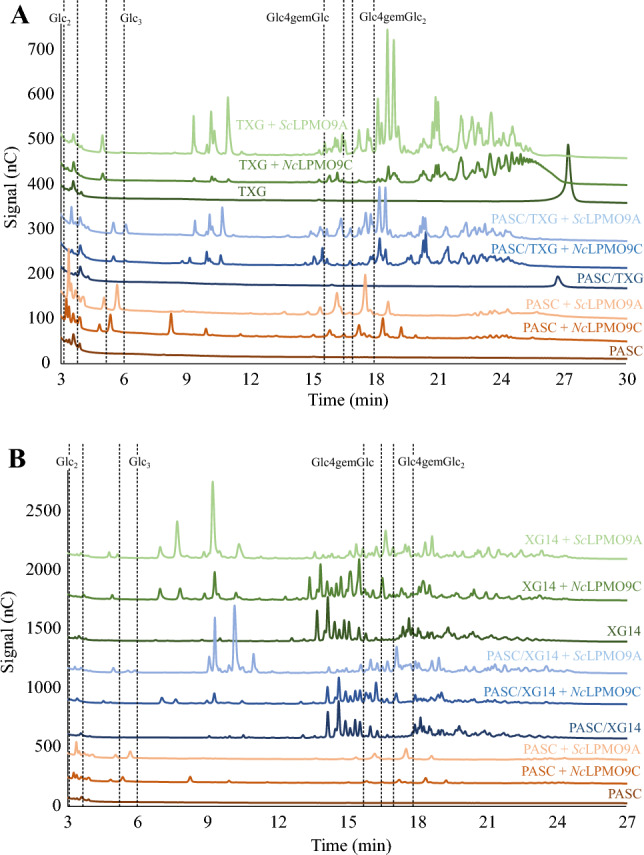
HPAEC-PAD chromatograms for reactions with *Sc*LPMO9A or *Nc*LPMO9C and PASC, tamarind xyloglucan (TXG), xyloglucan tetradecamer (XG14), or mixtures of PASC and TXG or XG14. Panel A shows reactions with PASC and TXG, while Panel B shows reactions with PASC and XG14. Reaction set-ups are indicated directly above the chromatograms in colors corresponding to the relevant chromatogram. Reactions contained 1 µM LPMO, 1 mM AscA, and either 2 g/L TXG or XG14, or, in the reactions containing hemicellulosic substrate and PASC, 2 g/L of each substrate (4 g/L total substrate concentration). Reactions with PASC alone contained 4 g/L PASC. Reactions lacking LPMO contained the aforementioned concentrations of substrate and 1 mM AscA. Reactions were incubated in 50 mM BisTris-HCl pH 6.5 at 40 °C and 1000 rpm for 16 h. Key products derived from LPMO activity on PASC (Glc_2_, Glc_3_, Glc4gemGlc, Glc4gemGlc_2_) are indicated within dashed rectangles (note that there is a slight shift between the PASC + *Sc*LPMO9A and the PASC + *Nc*LPMO9C chromatograms). Control reactions in the absence of AscA did not show any product formation for either LPMO. All reactions were carried out in triplicate and gave similar product profiles.

HPAEC-PAD chromatograms for reactions with TXG (Fig. 7A) showed clear activity of *Sc*LPMO9A and *Nc*LPMO9C, both in reactions with PASC/TXG and reactions with TXG alone. *Sc*LPMO9A seemed to generate more products than *Nc*LPMO9C, especially in reactions with only TXG. Overall, the product patterns of the two enzymes look similar and these patterns resemble those generated by previously described LPMOs (including *Nc*LPMO9C) that act on xyloglucan and that are “substitution-sensitive,” where the latter means that they only, or primarily, cleave the glucan chain at a non-substituted glucose^77–79^. Still, Fig. 7A shows minor differences in the product spectra of the two LPMOs, and differences were also observed when analyzing reactions with a mixture of xyloglucan tetradecamer, XG14 (Fig. 7B). Thus, the two enzymes do display different cleavage preferences when acting on xyloglucan.

MALDI-TOF MS analysis of products generated in the reaction with TXG confirmed that *Sc*LPMO9A is substitution-sensitive, since all abundant products contained a multitude of three pentoses (see Fig. S1 and its legend). For example, the mass spectrum for the various tetrameric products (Fig. S1) showed xyloglucan-derived oxidized species differing by *m/z* 162 and all containing 3 pentoses (*m/z* 132), corresponding to the oxidized non-hydrated keto species and the hydrated geminal diol species of xyloglucan fragments GXXX, GXXL, and GXLL (where G is a β-1,4-linked d-glucosyl unit, X is a glucosyl substituted with an α-1,6-linked d-xylosyl, and L corresponds to X but with a further substitution of the xylosyl with a β-1,2-linked d-galactosyl, according to standard xyloglucan nomenclature^80^). This TXG product pattern resembles what has previously been observed for *Nc*LPMO9C acting on xyloglucan^65,79^. If *Sc*LPMO9A would be able to cleave next to substituted sugars, other products would also have been observed in the spectrum shown in Fig. S1, such as at *m/z* 951 (4 hexoses, 2 pentoses) and *m/z* 1539 (6 hexoses, 4 pentoses), as has indeed been observed for TXG-active LPMOs that are less substitution-sensitive^78,79^.

MALDI-TOF MS analysis of products generated by *Sc*LPMO9A in the reaction with XG14 (Fig. S2) showed an accumulation of native and oxidized products, including the native XXX (*m/z* 923), XXXG (m/z 1085), XXLG (*m/z* 1247), and XLLG (*m/z* 1409), and oxidized XXLG (*m/z* 1245/1263) and XLLG (*m/z* 1407/1425). This pattern resembles what has previously been shown for *Nc*LPMO9C^65,81^, and confirms that, like *Nc*LPMO9C, *Sc*LPMO9A cleaves the xyloglucan backbone primarily adjacent to non-substituted glycosyl units.

Screening of *Sc*LPMO9A activity on beechwood xylan, birchwood xylan, and wheat arabinoxylan in combination with PASC, using MALDI-TOF MS for product detection, showed products identical to those observed in reactions with only PASC, while reactions with the xylan substrates alone showed no product formation. Despite differing from *Ls*LPMO9A (for which weak xylan activity has been reported) in only one of the three substrate-binding residues purported to contribute to xylanolytic activity, and despite this difference being minimal (Asn → Asp), *Sc*LPMO9A did not show reductant-dependent oxidative activity towards xylan.

### Synergy with cellulases

The contribution of LPMOs to the saccharification of cellulose, including cellulose in sulfite-pulped spruce, is well-documented. LPMO-containing cellulase cocktails work better under conditions that promote LPMO activity^36,56^, while addition of LPMOs improves the saccharification power of LPMO-poor cellulase cocktails^38,82^. Interestingly, saccharification reactions with sulfite-pulped spruce, under conditions previously used to reveal the impact of cellulose-active LPMOs, showed that *Sc*LPMO9A did not boost cellulose hydrolysis by an LPMO-poor cellulase cocktail (Fig. S3A). The reaction with AscA and the LPMO did show some LPMO product formation (Fig. S3B), but the glucose production was decreased rather than increased, probably due to the lower cellulase content of this reaction. While higher than in reactions without supplemented LPMO, Glc4gemGlc product levels for the reaction with *Sc*LPMO9A were low compared to what one would expect for a truly cellulose-active LPMO (e.g. Müller et al.^36^) and decreased over time, which is due to product instability and indicates that LPMO activity had already stopped at the first measuring point, indicative of limited substrate availability. Considering the results described above, indicating that *Sc*LPMO9A only acts on soluble and amorphous substrates, it is likely that these low levels of LPMO products result from action on amorphous subfractions of the material, the degradation of which does not affect overall saccharification efficiency.

### Effect of H_2_O_2_ on oxidized product formation from PASC and cellopentaose

**Figure 8:**
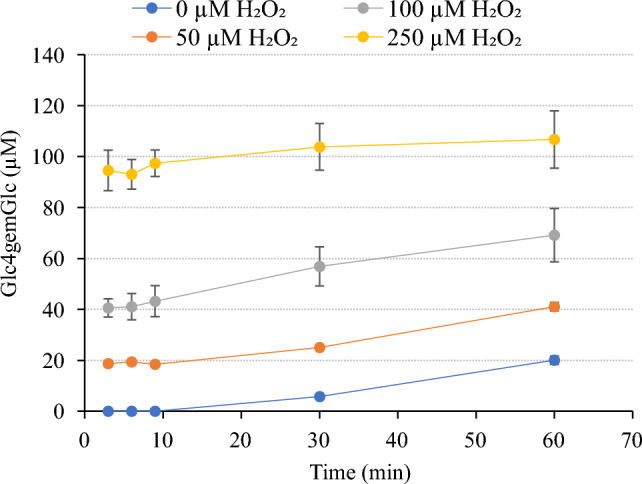
Effect of H_2_O_2_ on Glc4gemGlc production by *Sc*LPMO9A in reactions with PASC. The figure shows the production of Glc4gemGlc by 1 µM *Sc*LPMO9A in reactions containing 2 g/L PASC, 1 mM AscA, and different initial concentrations of supplemented H_2_O_2_ (0, 50, 100, or 250 µM). Reactions were performed in 50 mM Tris–HCl pH 7.5 at 45 °C and 1000 rpm. Control reactions lacking AscA did not show any formation of Glc4gemGlc. Error bars represent standard deviation between triplicates.

It is now well-established that LPMOs preferentially utilize H_2_O_2_ as a co-substrate to cleave glycosidic bonds and that the resulting peroxygenase reaction is fast^10,23,25^. In fact, a recent study of *Ls*LPMO9A, a close relative of *Sc*LPMO9A, concluded that this enzyme is unable to cleave glycosidic bonds in the absence of H_2_O_2_^26^. However, in the absence of substrate, reactions of the reduced LPMO with H_2_O_2_ can lead to auto-catalytic oxidation of the enzyme^10,83,84^. To assess the ability of *Sc*LPMO9A to productively use H_2_O_2_, we tested the effect of different initial concentrations of exogenously supplied H_2_O_2_ on the activity of *Sc*LPMO9A on PASC (Fig. 8). The formation of oxidized products was assessed by quantifying Glc4gemGlc, which can be quantified with reasonable accuracy^36,85^; other oxidized products, in particular the other primary soluble product, Glc4gemGlc_2_, and insoluble products (oxidations remaining on the fiber), were not quantified.

Without addition of H_2_O_2_, accumulation of Glc4gemGlc happened at a rate in the order of 0.3 min^−1^ (estimated from the progress curve in Fig. 8). Assuming that Glc4gemGlc represents about 40% of LPMO cleavages (see below for justification), this means that the LPMO operated at a rate in the order of 0.8 min^−1^. Such low rates are commonly observed for LPMOs in AscA-driven reactions^4^. Addition of H_2_O_2_ led to a dramatic increase in reaction speed: at the first measuring point, after 3 min, Glc4gemGlc levels amounted to approximately 40% of the added H_2_O_2_ for all three levels of inclusion. The progress curves starting at 3 min show slopes quite similar to the curve for the reaction with AscA only. This clearly shows that all H_2_O_2_ was consumed after 3 min and that the rest of the reaction was AscA-driven. The fact that the levels of Glc4gemGlc after 3 min amounted to 40% of added H_2_O_2_ could be taken to indicate stoichiometric conversion of H_2_O_2_, since other soluble oxidized products and insoluble oxidized products (i.e., oxidized sites remaining in the insoluble substrate) were not monitored and could very well amount to the other 60%. As a rule of thumb, one would expect some 50% of oxidized products to remain in the insoluble substrate in reactions with an LPMO that does not carry a CBM^86^, although this likely will vary between LPMOs. In any case, the levels of Glc4gemGlc show that a major fraction of the added H_2_O_2_ is used productively, and these levels are compatible with productive conversion being close to stoichiometric.

Based on the 3 min time point for the reaction with the highest H_2_O_2_ concentration, 250 µM, and considering formation of Glc4gemGlc only, the enzyme operated with a rate of at least some 80 min^−1^, which is two orders of magnitude higher compared to the reaction with AscA and no added H_2_O_2_. Notably, the progress curve for this reaction shows signs of enzyme inactivation, since the slope of the curve after 3 min is lower compared to the other progress curves.

**Figure 9:**
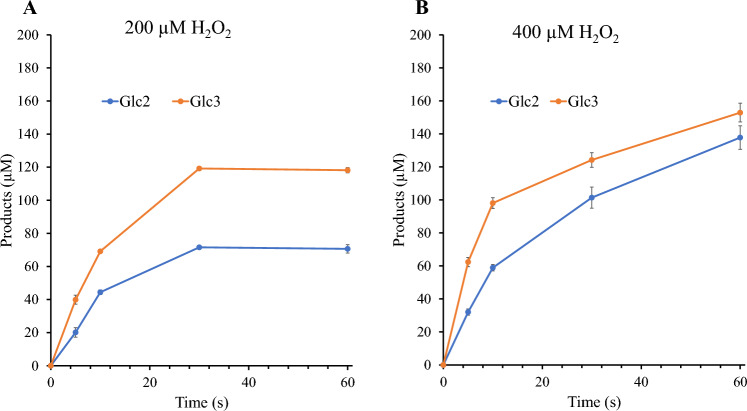
Peroxygenase activity of *Sc*LPMO9A acting on cellopentaose. The figure shows time courses for product formation in reactions containing 1 µM *Sc*LPMO9A, 50 µM AscA, 1 mM cellopentaose, and 200 µM (Panel A) or 400 µM (Panel B) H_2_O_2_. Reactions were performed in 50 mM sodium acetate pH 5.0 and were incubated at 40 °C and 500 rpm. Note that cleavage of cellopentaose leads either to a dimeric or a trimeric product; for example, in panel A, at 30 s, approximately 190 µM of cellopentaose has been converted, resulting in 70 µM of dimer and 120 µM of trimer. Samples were taken at 5, 10, 30, and 60 s. Error bars represent standard deviation between triplicates.

Rieder et al*.* have shown that when supplied with H_2_O_2_ and a soluble substrate, *Nc*LPMO9C is a very efficient peroxygenase, reaching catalytic rates above 100 s^−1^ and with the ability to productively use large amounts of H_2_O_2_ to stoichiometrically degrade the cello-oligomer substrate^25^. Rates well above 10 s^−1^ have also been reported for similar reactions with *Ls*LPMO9A^25,26^. Figure 9 shows progress curves for one-minute reactions of *Sc*LPMO9A with cellopentaose at two initial H_2_O_2_ concentrations. Conversion of cellopentaose was quantified by monitoring the generation of cellobiose and cellotriose, the amounts of which are expected to be equimolar to the amounts of oxidized trimer and dimer, respectively, although this equimolarity has been questioned in a recent study^26^. When supplemented with 200 µM H_2_O_2_ (Fig. 9A), near complete, seemingly almost stoichiometric conversion of H_2_O_2_ was achieved within 30 s, with product levels amounting to 190 µM. The actual level of LPMO-catalyzed cleavages may be somewhat lower because, under these conditions, some of the oxidized trimer may be spontaneously converted to the native dimer^72^ (this effect is more prominent at higher H_2_O_2_ concentrations, as is indeed visible in Fig. 9B). Based on the first 10 s of the experiment, *Sc*LPMO9A reached a rate of at least 11 s^−1^. When the H_2_O_2_ concentration was increased to 400 µM (Fig. 9B), *Sc*LPMO9A generated slightly less than 300 µM product in 1 min. Although initial rates appeared higher than when supplemented with 200 µM H_2_O_2_ (at least 15 s^−1^), under these conditions full conversion of H_2_O_2_ was not observed, and the reaction showed signs of LPMO inactivation and/or reductant depletion.

### Binding interactions by NMR

**Figure 10:**
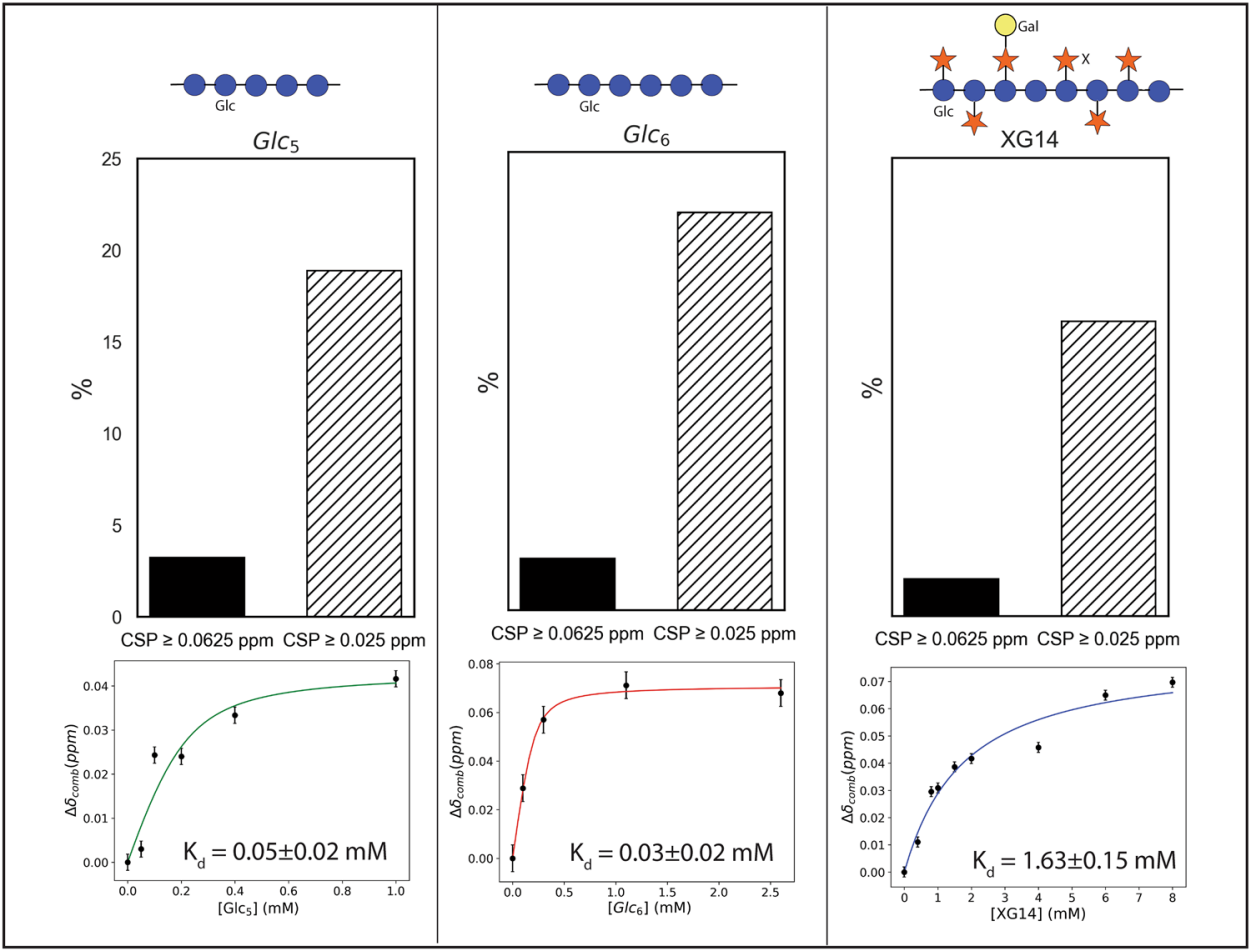
Binding interactions between *Sc*LPMO9A and cellopentaose (Glc_5_), cellohexaose (Glc_6_), and xyloglucan tetradecamer (XG14). Binding interactions between the substrates and the LPMO were measured by the number of backbone ^1^H-^15^N signals showing significant chemical shift perturbations (CSP ≥ 0.025 ppm), and perturbations corresponding to direct interactions (CSP ≥ 0.063 ppm) when titrated with the substrates. The dissociation constant (K_d_) of the interactions was calculated by plotting the combined CSP (Δδ_comb_ in ppm) against the substrate concentration (mM). Schematic presentations of Glc_5_, Glc_6_, and XG14 are placed above the plots. β-1,4-linked glucose monomers are shown as blue circles, α-1,6-linked xylosyl substitutions are shown as orange stars, and β-1,2-linked galactosyl substitutions are shown as yellow circles. Note that XG14 is a mixture of oligomers and that the number and positions of xylosyl and galactosyl substitutions are unknown.

**Figure 11:**
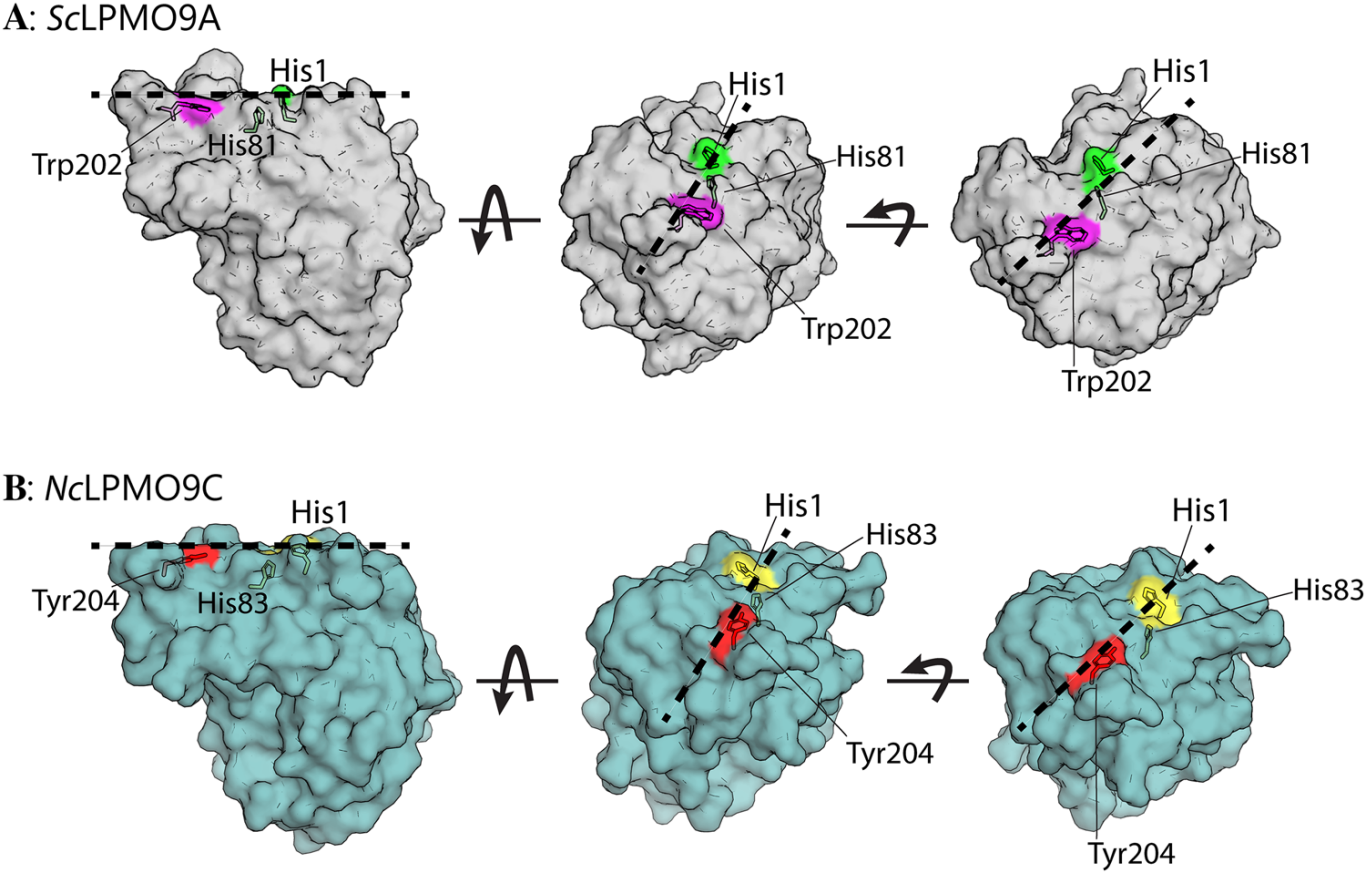
Substrate-binding surface topology of *Sc*LPMO9A and *Nc*LPMO9C. The figure shows the substrate-binding surface topology of an AlphaFold model of *Sc*LPMO9A (Panel A) and an X-ray crystal structure of *Nc*LPMO9C (PDB ID 4D7U; Panel B). The side chains of the copper-binding histidines and a conserved surface-exposed aromatic residue are shown as colored sticks. The black dashed line illustrates how a linear polysaccharide chain could interact with the substrate-binding surface of the LPMOs.

Interactions between copper-free, *apo*-*Sc*LPMO9A and cellopentaose, cellohexaose, and XG14 were probed by following Chemical Shift Perturbations (CSPs) in ^15^N-HSQCs during titrations of the protein with the individual oligosaccharides. Based on the results, the dissociation constants (K_d_) for the three substrates were found to have the following order: cellohexaose (in the range of 0.01–0.04 mM) < cellopentaose (in the range of 0.03–0.05 mM) < XG14 (in the range of 1.63–3.55 mM) (Figs. 10 and S4). This trend in binding affinities differs from observations made for the* apo*-form of *Nc*LPMO9C, which has a higher affinity for the branched XG14 (0.420 ± 0.02 mM) than for Glc_6_ (1.1 ± 0.1 mM)^69^. Differences in binding affinities between the two LPMOs can, in part, be explained by variations in their substrate-binding surfaces: the AlphaFold model of *Sc*LPMO9A shows a shallow groove, whereas the surface of *Nc*LPMO9C is flatter (Fig. 11). A groove-like surface is well-compatible with binding of linear substrates, while binding of branched substrates, such as XG14, may be sterically hindered. It is conceivable that such branched substrates would bind better to *Nc*LPMO9C, which has a flatter surface, as has indeed been observed experimentally.

In the recorded ^15^N-HSQC spectra, the ^1^H-^15^N signals of the residues involved in substrate binding are expected to change as the substrate concentration increases during the titration. The ^15^N-HSQC spectrum can thus provide quantitative information about the number of residues involved in substrate interaction, even without a resonance assignment linking the ^1^H-^15^N signal to the primary structure of the protein. The largest changes in CSP values are expected for residues directly involved in substrate binding, while residues farther away from the substrate-binding surface typically display smaller changes or no change ^67^.

In response to titration with cellohexaose, 54 ^1^H-^15^N signals showed a CSP ≥ 0.025 ppm, while seven ^1^H-^15^N signals were significantly perturbed with a CSP ≥ 0.063 ppm, indicating that the corresponding seven residues likely have a direct interaction with cellohexaose (Fig. 10). For the interaction between cellopentaose and *Sc*LPMO9A, a slightly lower number (47) ^1^H-^15^N signals showed a CSP ≥ 0.025 ppm, while in this case nine ^1^H-^15^N signals displayed a CSP ≥ 0.063 ppm. The changes in CSP showed that the interaction with XG14 involves fewer residues, with 40 ^1^H-^15^N signals having a CSP ≥ 0.025 ppm and only five ^1^H-^15^N signals showing a direct (CSP ≥ 0.063 ppm) interaction with XG14. Titration experiments by NMR with *Nc*LPMO9C have previously demonstrated that XG14 binds to a larger area (i.e., interacting with a higher number of residues) compared to cellohexaose^69^, further underpinning differences between this enzyme and *Sc*LPMO9A.

In addition to providing a picture of the number of residues involved in interacting with each substrate, data from ^15^N-HSQC spectra also show whether the same or different residues are involved in binding the different substrates (Fig. S5). Comparison of the ^1^H-^15^N signals affected by titrations with cellohexaose and cellopentaose shows that some 60% of affected signals are the same, both when looking at CSP ≥ 0.025 ppm and CSP ≥ 0.063 ppm (Fig. S5). The differences between the two substrates may be due to size differences and to the occurrence of different binding modes with similar affinities. Comparison of the cello-oligomers with XG14 showed much larger differences, with only about 35% of the affected ^1^H-^15^N signals being the same when considering CSP ≥ 0.025 ppm. For residues with CSP ≥ 0.063 ppm, the difference between the cello-oligomers and XG14 was even bigger (Fig. S5). Thus, it would seem that binding of XG14 is quite different from binding of cello-oligomers, with, as shown above, the latter being the preferred substrates of *Sc*LPMO9A.

**Figure 12:**
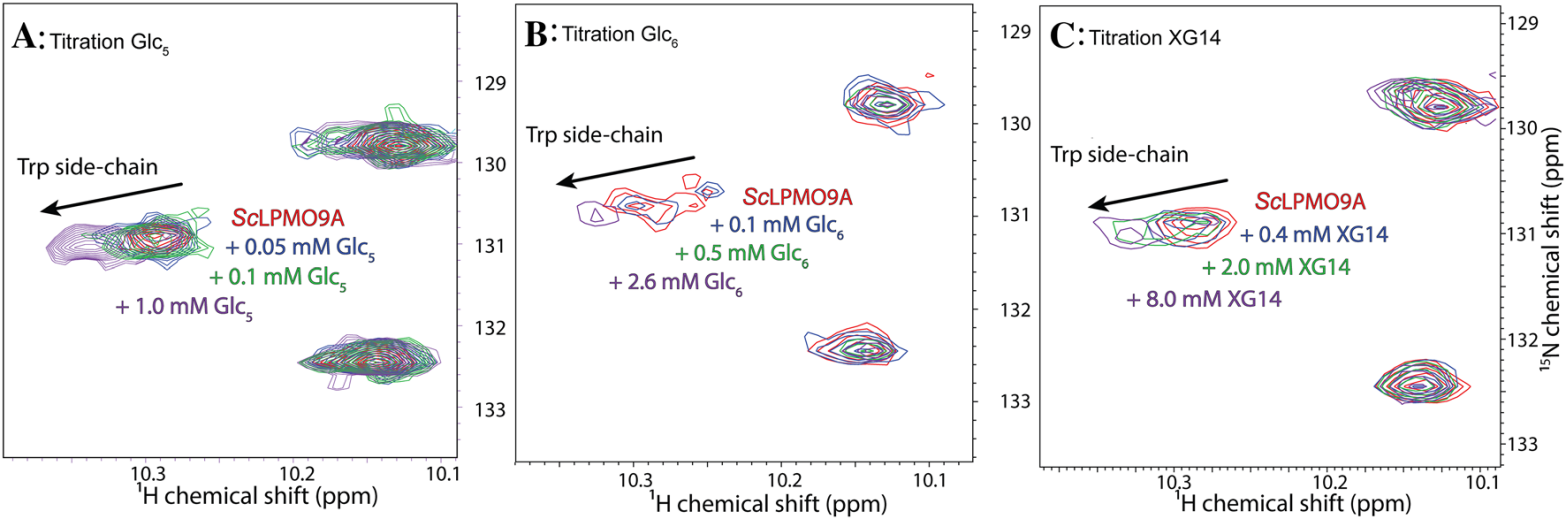
Changes in the chemical shift of a tryptophan residue (H^N^: 10.294, N = 130.890) upon titration with the three substrates cellopentaose (Glc_5_), cellohexaose (Glc_6_), and xyloglucan tetradecamer (XG14). Panel A: Overlay of ^15^N-HSQC spectra recorded when *Sc*LPMO9A was titrated with 0.05, 0.1, and 1.0 mM cellopentaose. Panel B: Overlay of ^15^N-HSQC spectra recorded when *Sc*LPMO9A was titrated with 0.1, 0.5, and 2.6 mM cellohexaose. Panel C: Overlay of ^15^N-HSQC spectra recorded when *Sc*LPMO9A was titrated with 0.4, 2.0, and 8.0 mM XG14. Arrows indicate the direction of the change in chemical shift in all three panels.

Interestingly, a ^1^H-^15^N signal with a chemical shift fitting the side chain of a tryptophan residue (chemical shift of H^N^: 10.29 ppm, N:130.9 ppm) displayed a CSP of 0.041 ppm, 0.040 ppm, and 0.050 ppm when titrated with cellopentaose, cellohexaose, and XG14, respectively (Fig. 12). These CSPs indicate that a tryptophan is a part of the binding surface for all three substrates tested, however, without a chemical shift assignment it is not possible to determine if this is the side chain of Trp202, which, as shown in Fig. 3, likely interacts with the substrate. The favorable CH-π interactions between sugar rings and the side chain of tryptophan^87^ could explain the high affinity of *Sc*LPMO9A towards both cellopentaose and cellohexaose compared with *Nc*LPMO9C, which carries a tyrosine at this position^69^ (Fig. 12).

## Concluding remarks

The data presented in this study show that *Sc*LPMO9A is a C4-oxidizing LPMO with activity on amorphous cellulose, soluble cello-oligosaccharides, and various hemicellulose glycans, and with limited ability to contribute to the saccharification of crystalline cellulose. The complete degradation of 1 mM cellotetraose under the conditions tested is of particular interest, since activity on this substrate among cello-oligomer-degrading LPMOs tends to be low^7,30^. Further in-depth analysis of the substrate-binding residues surrounding the active site of *Sc*LPMO9A, preferably based on crystal structures, is needed to explain the structural basis for the observed activity on cellotetraose.

Comparison of *Sc*LPMO9A and *Nc*LPMO9C in the degradation of glucomannan, mixed-linkage β-glucan, and xyloglucan showed that the enzymes share certain properties, such as both being substitution-sensitive in TXG degradation. The product profiles and substrate binding studies did show subtle differences indicating that *Sc*LPMO9A prefers linear over branched substrates. To the best of our knowledge, *Sc*LPMO9A is so far the LPMO with the highest affinity for soluble natural substrates like cellohexaose, with a *K*_d_ that is an order of magnitude smaller than for *Nc*LPMO9C^69^.

During the course of this study, a study containing comparative functional data for eight fungal C4-oxidizing LPMOs, including *Sc*LPMO9A, was published^7^. All these LPMOs were expressed in the yeast *Pichia pastoris* and shown to be active on cello-oligomers and/or hemicellulosic glycans, albeit with seemingly different efficiencies. The conclusions of the aforementioned study regarding the presumed preferred binding mode for cellohexaose are compatible with our observations. However, remarkably, while some of the LPMOs described in this study seemed to show a substrate spectrum similar to the two LPMOs studied above, Frandsen et al*.* concluded that *Sc*LPMO9A is not active on glucomannan and TXG, nor on cellotetraose. The conclusions of the present study are different. Of note, proteomics analysis (see Materials and methods) showed that the enzyme used in our study was indeed *Sc*LPMO9A (UniProt ID D8Q364).

The discovery that LPMOs may use H_2_O_2_ rather than O_2_ to break down glycosidic bonds has created some controversy, but has also shown that LPMOs are faster enzymes than originally believed. Although *Sc*LPMO9A appears to be more sensitive to H_2_O_2_ than *Nc*LPMO9C under the conditions tested in the present study, *Sc*LPMO9A still uses H_2_O_2_ very efficiently when acting on cellopentaose, reaching a rate of approx. 11 s^−1^ when supplied with 200 µM H_2_O_2_. We also show that *Sc*LPMO9A readily uses H_2_O_2_ to degrade PASC, reaching rates in the order of at least several per second rather than about 1 min^−1^. To the best of our knowledge, this is the first time that such a high LPMO activity is demonstrated on this much used amorphous cellulosic substrate.

All in all, *Sc*LPMO9A seems specifically tailored to work on amorphous and soluble substrates. Given that powerful hydrolytic cellulases co-secreted with LPMOs in natural biomass-degrading ecosystems readily degrade soluble oligosaccharides, it is unlikely that fungi have evolved LPMOs with the specialized purpose of degrading these oligomers. Thus, it is conceivable that enzymes such as *Sc*LPMO9A play hitherto undiscovered roles in lignocellulose conversion, or perhaps even in the conversion of non-lignocellulosic substrates.

### Supplementary Information

Supplementary Figures.

## Data Availability

The data that support the findings of this study are available in Figs. 1–12 and in the Supporting Information file.

## References

[1] Vaaje-Kolstad G (2010). An oxidative enzyme boosting the enzymatic conversion of recalcitrant polysaccharides. Science.

[2] Hemsworth GR, Johnston EM, Davies GJ, Walton PH (2015). Lytic polysaccharide monooxygenases in biomass conversion. Trends Biotechnol..

[3] Johansen KS (2016). Discovery and industrial applications of lytic polysaccharide mono-oxygenases. Biochem. Soc. Trans..

[4] Bissaro B, Várnai A, Røhr ÅK, Eijsink VGH (2018). Oxidoreductases and reactive oxygen species in conversion of lignocellulosic biomass. Microbiol. Mol. Biol. Rev..

[5] Vandhana TM (2022). On the expansion of biological functions of lytic polysaccharide monooxygenases. New Phytol..

[6] Lenfant N (2017). A bioinformatics analysis of 3400 lytic polysaccharide oxidases from family AA9. Carbohydr. Res..

[7] Frandsen KEH (2021). Identification of the molecular determinants driving the substrate specificity of fungal lytic polysaccharide monooxygenases (LPMOs). J. Biol. Chem..

[8] Tõlgo M (2022). Comparison of six lytic polysaccharide monooxygenases from Thermothielavioides terrestris shows that functional variation underlies the multiplicity of LPMO genes in filamentous fungi. Appl. Environ. Microbiol..

[9] Phillips CM, Beeson WT, Cate JH, Marletta MA (2011). Cellobiose dehydrogenase and a copper-dependent polysaccharide monooxygenase potentiate cellulose degradation by Neurospora crassa. ACS Chem. Biol..

[10] Bissaro B (2017). Oxidative cleavage of polysaccharides by monocopper enzymes depends on H_2_O_2_. Nat. Chem. Biol..

[11] Jones SM (2020). Kinetic analysis of amino acid radicals formed in H_2_O_2_-driven Cu^I^ LPMO reoxidation implicates dominant homolytic reactivity. Proc. Natl. Acad. Sci. U. S. A..

[12] Quinlan RJ (2011). Insights into the oxidative degradation of cellulose by a copper metalloenzyme that exploits biomass components. Proc. Natl. Acad. Sci. U. S. A..

[13] Vaaje-Kolstad G (2017). Structural diversity of lytic polysaccharide monooxygenases. Curr. Opin. Struct. Biol..

[14] Westereng B (2015). Enzymatic cellulose oxidation is linked to lignin by long-range electron transfer. Sci. Rep..

[15] Kracher D (2016). Extracellular electron transfer systems fuel cellulose oxidative degradation. Science.

[16] Frommhagen M, Westphal AH, van Berkel WJH, Kabel MA (2018). Distinct substrate specificities and electron-donating systems of fungal lytic polysaccharide monooxygenases. Front. Microbiol..

[17] Chalak A (2019). Influence of the carbohydrate-binding module on the activity of a fungal AA9 lytic polysaccharide monooxygenase on cellulosic substrates. Biotechnol. Biofuels.

[18] Beeson WT, Phillips CM, Cate JH, Marletta MA (2012). Oxidative cleavage of cellulose by fungal copper-dependent polysaccharide monooxygenases. J. Am. Chem. Soc..

[19] Hedison TM (2021). Insights into the H_2_O_2_-driven catalytic mechanism of fungal lytic polysaccharide monooxygenases. FEBS J..

[20] Kittl R (2012). Production of four Neurospora crassa lytic polysaccharide monooxygenases in Pichia pastoris monitored by a fluorimetric assay. Biotechnol. Biofuels.

[21] Stepnov AA, Eijsink VGH, Forsberg Z (2022). Enhanced in situ H_2_O_2_ production explains synergy between an LPMO with a cellulose-binding domain and a single-domain LPMO. Sci. Rep..

[22] Hangasky JA, Iavarone AT, Marletta MA (2018). Reactivity of O_2_ versus H_2_O_2_ with polysaccharide monooxygenases. Proc. Natl. Acad. Sci. U. S. A..

[23] Kuusk S (2018). Kinetics of H_2_O_2_-driven degradation of chitin by a bacterial lytic polysaccharide monooxygenase. J. Biol. Chem..

[24] Kont R, Bissaro B, Eijsink VGH, Väljamäe P (2020). Kinetic insights into the peroxygenase activity of cellulose-active lytic polysaccharide monooxygenases (LPMOs). Nat. Commun..

[25] Rieder L, Stepnov AA, Sørlie M, Eijsink VGH (2021). Fast and specific peroxygenase reactions catalyzed by fungal mono-copper enzymes. Biochemistry.

[26] Brander S (2021). Scission of glucosidic bonds by a Lentinus similis lytic polysaccharide monooxygenases is strictly dependent on H_2_O_2_ while the oxidation of saccharide products depends on O_2_. ACS Catal..

[27] Drula E (2022). The carbohydrate-active enzyme database: Functions and literature. Nucleic Acids Res..

[28] Levasseur A (2013). Expansion of the enzymatic repertoire of the CAZy database to integrate auxiliary redox enzymes. Biotechnol. Biofuels.

[29] Petrović DM (2018). Methylation of the N-terminal histidine protects a lytic polysaccharide monooxygenase from auto-oxidative inactivation. Protein Sci..

[30] Isaksen T (2014). A C4-oxidizing lytic polysaccharide monooxygenase cleaving both cellulose and cello-oligosaccharides. J. Biol. Chem..

[31] Borisova AS (2015). Structural and functional characterization of a lytic polysaccharide monooxygenase with broad substrate specificity. J. Biol. Chem..

[32] Frandsen KEH (2016). The molecular basis of polysaccharide cleavage by lytic polysaccharide monooxygenases. Nat. Chem. Biol..

[33] Simmons TJ (2017). Structural and electronic determinants of lytic polysaccharide monooxygenase reactivity on polysaccharide substrates. Nat. Commun..

[34] Tandrup T (2020). Oligosaccharide binding and thermostability of two related AA9 lytic polysaccharide monooxygenases. Biochemistry.

[35] Cannella D, Hsieh C-WC, Felby C, Jørgensen H (2012). Production and effect of aldonic acids during enzymatic hydrolysis of lignocellulose at high dry matter content. Biotechnol. Biofuels.

[36] Müller G (2018). The impact of hydrogen peroxide supply on LPMO activity and overall saccharification efficiency of a commercial cellulase cocktail. Biotechnol. Biofuels.

[37] Harris PV (2014). New enzyme insights drive advances in commercial ethanol production. Curr. Opin. Chem. Biol..

[38] Müller G (2015). Harnessing the potential of LPMO-containing cellulase cocktails poses new demands on processing conditions. Biotechnol. Biofuels.

[39] Costa THF (2020). Demonstration-scale enzymatic saccharification of sulfite-pulped spruce with addition of hydrogen peroxide for LPMO activation. Biofuels, Bioprod. Biorefining.

[40] Ohm RA (2010). Genome sequence of the model mushroom *Schizophyllum commune*. Nat. Biotechnol..

[41] Zhu N (2016). Comparative analysis of the secretomes of *Schizophyllum commune* and other wood-decay Basidiomycetes during solid-state fermentation reveals its unique lignocellulose-degrading enzyme system. Biotechnol. Biofuels.

[42] Almási É (2019). Comparative genomics reveals unique wood-decay strategies and fruiting body development in the Schizophyllaceae. New Phytol..

[43] Armougom F (2006). Expresso: Automatic incorporation of structural information in multiple sequence alignments using 3D-Coffee. Nucleic Acids Res..

[44] Larsson A (2014). AliView: A fast and lightweight alignment viewer and editor for large datasets. Bioinformatics.

[45] Darriba D, Taboada GL, Doallo R, Posada D (2011). ProtTest 3: Fast selection of best-fit models of protein evolution. Bioinformatics.

[46] Letunic I, Bork P (2007). Interactive Tree Of Life (iTOL): An online tool for phylogenetic tree display and annotation. Bioinformatics.

[47] Kelley LA (2015). The Phyre2 web portal for protein modeling, prediction and analysis. Nat. Protoc..

[48] Zhang W (2018). Development an effective system to expression recombinant protein in *E. coli* via comparison and optimization of signal peptides: expression of Pseudomonas fluorescens BJ-10 thermostable lipase as case study. Microb. Cell Factories.

[49] Courtade G (2017). A novel expression system for lytic polysaccharide monooxygenases. Carbohydr. Res..

[50] Manoil C, Beckwith J (1986). A genetic approach to analyzing membrane protein topology. Science.

[51] Gasteiger E, Walker JM (2005). Protein identification and analysis tools on the ExPASy server. The Proteomics Protocols Handbook.

[52] Loose JS (2014). A rapid quantitative activity assay shows that the Vibrio cholerae colonization factor GbpA is an active lytic polysaccharide monooxygenase. FEBS Lett..

[53] Diep DB (2007). Common mechanisms of target cell recognition and immunity for class II bacteriocins. Proc. Natl. Acad. Sci..

[54] Wood TM (1988). Preparation of crystalline, amorphous, and dyed cellulase substrates. Methods Enzymol..

[55] Rødsrud G, Lersch M, Sjöde A (2012). History and future of world's most advanced biorefinery in operation. Biomass Bioenergy.

[56] Chylenski P (2017). Enzymatic degradation of sulfite-pulped softwoods and the role of LPMOs. Biotechnol. Biofuels.

[57] Breslmayr E (2018). A fast and sensitive activity assay for lytic polysaccharide monooxygenase. Biotechnol. Biofuels.

[58] Aachmann FL (2012). NMR structure of a lytic polysaccharide monooxygenase provides insight into copper binding, protein dynamics, and substrate interactions. Proc. Natl. Acad. Sci. U. S. A..

[59] Sørlie M, Seefeldt LC, Parker VD (2000). Use of stopped-flow spectrophotometry to establish midpoint potentials for redox proteins. Anal. Biochem..

[60] Liu Y, Seefeldt LC, Parker VD (1997). Entropies of redox reactions between proteins and mediators: the temperature dependence of reversible electrode potentials in aqueous buffers. Anal. Biochem..

[61] Bradford MM (1976). A rapid and sensitive method for the quantitation of microgram quantities of protein utilizing the principle of protein-dye binding. Anal. Biochem..

[62] Westereng B (2017). Analyzing activities of lytic polysaccharide monooxygenases by liquid chromatography and mass spectrometry. Methods Mol. Biol..

[63] Hegnar OA (2021). Quantifying oxidation of cellulose-associated glucuronoxylan by two lytic polysaccharide monooxygenases from *Neurospora crassa*. Appl. Environ. Microbiol..

[64] Østby H (2022). Chromatographic analysis of oxidized cello-oligomers generated by lytic polysaccharide monooxygenases using dual electrolytic eluent generation. J. Chromatogr. A.

[65] Agger JW (2014). Discovery of LPMO activity on hemicelluloses shows the importance of oxidative processes in plant cell wall degradation. Proc. Natl. Acad. Sci. U. S. A..

[66] Teilum K, Kunze MBA, Erlendsson S, Kragelund BB (2017). (S)Pinning down protein interactions by NMR. Protein Sci..

[67] Williamson MP (2013). Using chemical shift perturbation to characterise ligand binding. Prog. Nucl. Magn. Reson. Spectrosc..

[68] Jagadeeswaran G, Gainey L, Mort AJ (2018). An AA9-LPMO containing a CBM1 domain in Aspergillus nidulans is active on cellulose and cleaves cello-oligosaccharides. AMB Express.

[69] Courtade G (2016). Interactions of a fungal lytic polysaccharide monooxygenase with β-glucan substrates and cellobiose dehydrogenase. Proc. Natl. Acad. Sci. U. S. A..

[70] Beeson WT (2015). Cellulose degradation by polysaccharide monooxygenases. Annu. Rev. Biochem..

[71] Frandsen KEH, Lo Leggio L (2016). Lytic polysaccharide monooxygenases: A crystallographer's view on a new class of biomass-degrading enzymes. IUCrJ.

[72] Westereng B (2016). Simultaneous analysis of C1 and C4 oxidized oligosaccharides, the products of lytic polysaccharide monooxygenases acting on cellulose. J. Chromatogr. A.

[73] Aldaeus F (2015). The supramolecular structure of cellulose-rich wood pulps can be a determinative factor for enzymatic hydrolysability. Cellulose.

[74] Frommhagen M (2015). Discovery of the combined oxidative cleavage of plant xylan and cellulose by a new fungal polysaccharide monooxygenase. Biotechnol. Biofuels.

[75] Petrović DM (2019). Comparison of three seemingly similar lytic polysaccharide monooxygenases from *Neurospora crassa* suggests different roles in plant biomass degradation. J. Biol. Chem..

[76] Eijsink VGH (2019). On the functional characterization of lytic polysaccharide monooxygenases (LPMOs). Biotechnol. Biofuels.

[77] Nekiunaite L (2016). *Fg*LPMO9A from *Fusarium graminearum* cleaves xyloglucan independently of the backbone substitution pattern. FEBS Lett..

[78] Monclaro AV (2020). Characterization of two family AA9 LPMOs from *Aspergillus tamarii* with distinct activities on xyloglucan reveals structural differences linked to cleavage specificity. PLoS One.

[79] Sun P (2020). Configuration of active site segments in lytic polysaccharide monooxygenases steers oxidative xyloglucan degradation. Biotechnol. Biofuels.

[80] Fry SC (1993). An unambiguous nomenclature for xyloglucan-derived oligosaccharides. Physiologia Plantarum.

[81] Kojima Y (2016). A lytic polysaccharide monooxygenase with broad xyloglucan specificity from the brown-rot fungus *Gloeophyllum trabeum* and its action on cellulose-xyloglucan complexes. Appl. Environ. Microbiol..

[82] Tuveng TR (2020). A thermostable bacterial lytic polysaccharide monooxygenase with high operational stability in a wide temperature range. Biotechnol. Biofuels.

[83] Kuusk S (2019). Kinetic insights into the role of the reductant in H_2_O_2_-driven degradation of chitin by a bacterial lytic polysaccharide monooxygenase. J. Biol. Chem..

[84] Kuusk S, Väljamäe P (2021). Kinetics of H_2_O_2_-driven catalysis by a lytic polysaccharide monooxygenase from the fungus *Trichoderma reesei*. J. Biol. Chem..

[85] Østby H (2022). Substrate-dependent cellulose saccharification efficiency and LPMO activity of Cellic CTec2 and a cellulolytic secretome from *Thermoascus aurantiacus* and the impact of H_2_O_2_-producing glucose oxidase. ACS Sustain. Chem. Eng..

[86] Courtade G (2018). The carbohydrate-binding module and linker of a modular lytic polysaccharide monooxygenase promote localized cellulose oxidation. J. Biol. Chem..

[87] Hudson KL (2015). Carbohydrate–aromatic interactions in proteins. J. Am. Chem. Soc..

[88] Wu M (2013). Crystal structure and computational characterization of the lytic polysaccharide monooxygenase GH61D from the Basidiomycota fungus *Phanerochaete chrysosporium*. J. Biol. Chem..

[89] Laurent CVFP (2019). Influence of lytic polysaccharide monooxygenase active site segments on activity and affinity. Int. J. Mol. Sci..

