# Supplementary material for: Functional characterization of a lytic polysaccharide monooxygenase from Schizophyllum commune that degrades non-crystalline substrates
Source: Sci Rep. 2023 Oct 13;13:17373. doi: 10.1038/s41598-023-44278-1 (PMC10575960; doi:10.1038/s41598-023-44278-1)
Supplement: Supplementary file 1 — Supplementary Figures. [file 41598_2023_44278_MOESM1_ESM.docx]

**Functional characterization of a lytic polysaccharide monooxygenase from *Schizophyllum commune* that degrades non-crystalline substrates**

Heidi Østby^1^, Idd A. Christensen^2^, Karen Hennum^1^, Anikó Várnai^1^, Edith Buchinger^2^, Siri Grandal^2^, Gaston Courtade^2^, Olav A. Hegnar^1^, Finn L. Aachmann^2^, and Vincent G. H. Eijsink^1,*^

^1^Norwegian University of Life Sciences (NMBU), Faculty of Chemistry, Biotechnology, and Food Science, P.O. Box 5003, N-1432 Ås, Norway
^2^NTNU Norwegian University of Science and Technology, Norwegian Biopolymer Laboratory (NOBIPOL), Department of Biotechnology and Food Science, Sem Sælands vei 6/8, N-7491 Trondheim, Norway

**^*^**Address correspondence to Vincent G. H. Eijsink, vincent.eijsink@nmbu.no
Norwegian University of Life Sciences (NMBU), Faculty of Chemistry, Biotechnology, and Food Science, P.O. Box 5003, N-1432 Ås, Norway, Telephone: +47 67232463.

**Supporting Information**


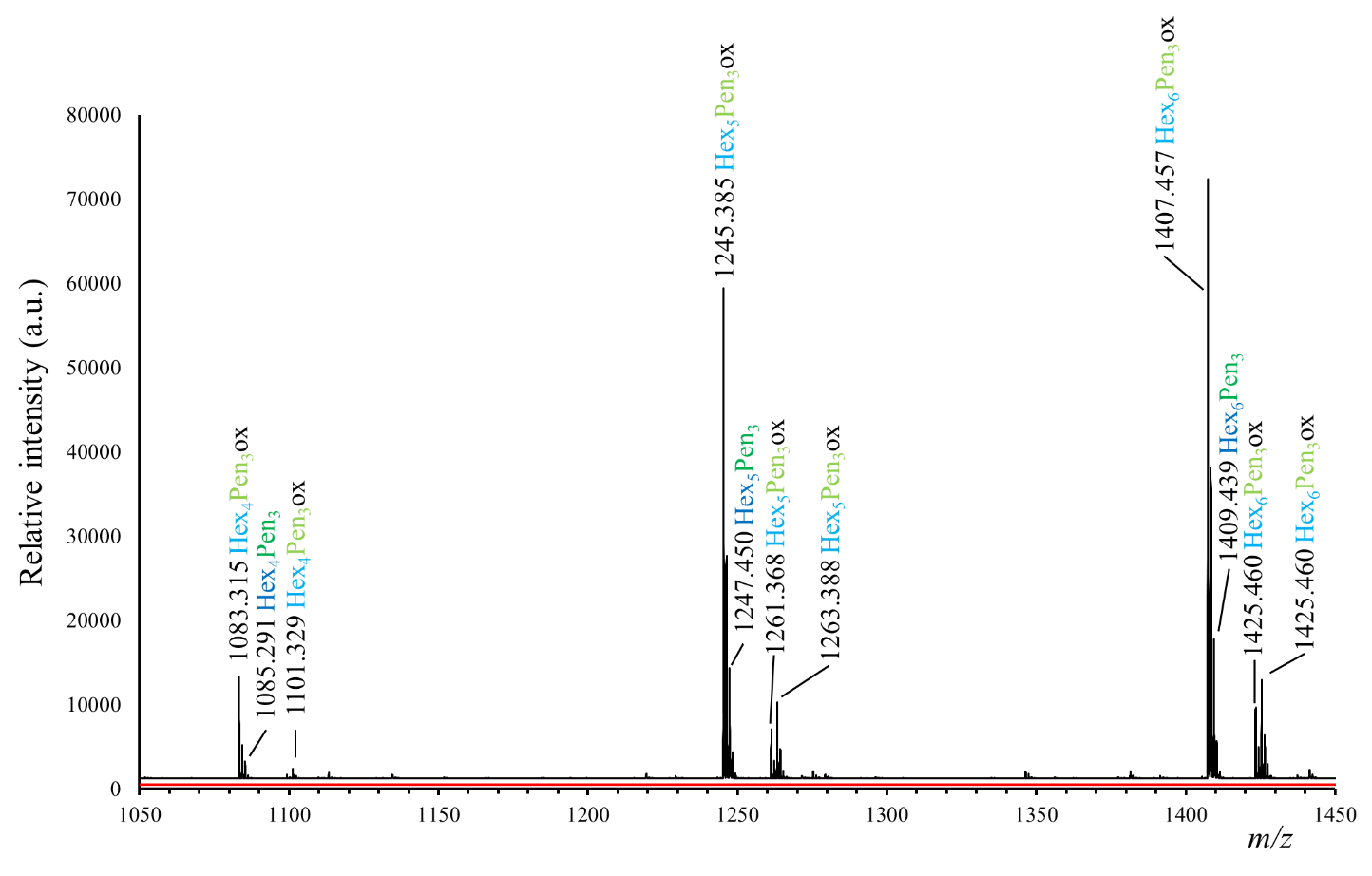
**Figure S1. MALDI-TOF MS analysis of products generated in reactions of *Sc*LPMO9A with TXG in the presence of AscA.** The spectrum shows the samples analyzed with HPAEC-PAD in **Figure 7A** in the main text. The red spectrum shows the corresponding reaction without AscA. The labeled products are the sodium adducts of native species (e.g. *m/z* 1085.3), oxidized species in the non-hydrated keto form (e.g. *m/z* 1083.3), and, to a lesser extent, the corresponding geminal diol form (e.g. *m/z* 1101.3), for Hex_4_Pen_3_ (XXXG), Hex_5_Pen_3_ (XXLG), and Hex_6_Pen_3_ (XLLG), where G is glucose, X is glucose substituted with xylose, and L is X substituted with galactose. Note that the positions of the various main chain units (G, X, L) cannot be derived from the MS data. The absence of fragments containing less or more than three pentoses (e.g. *m/z* 951 for Hex_4_Pen_2_, or *m/z* 1539 for Hex_6_Pen_4_; not shown within the range of the figure) indicates that the LPMO only cleaves the main chain of TXG at unsubstituted glucoses.


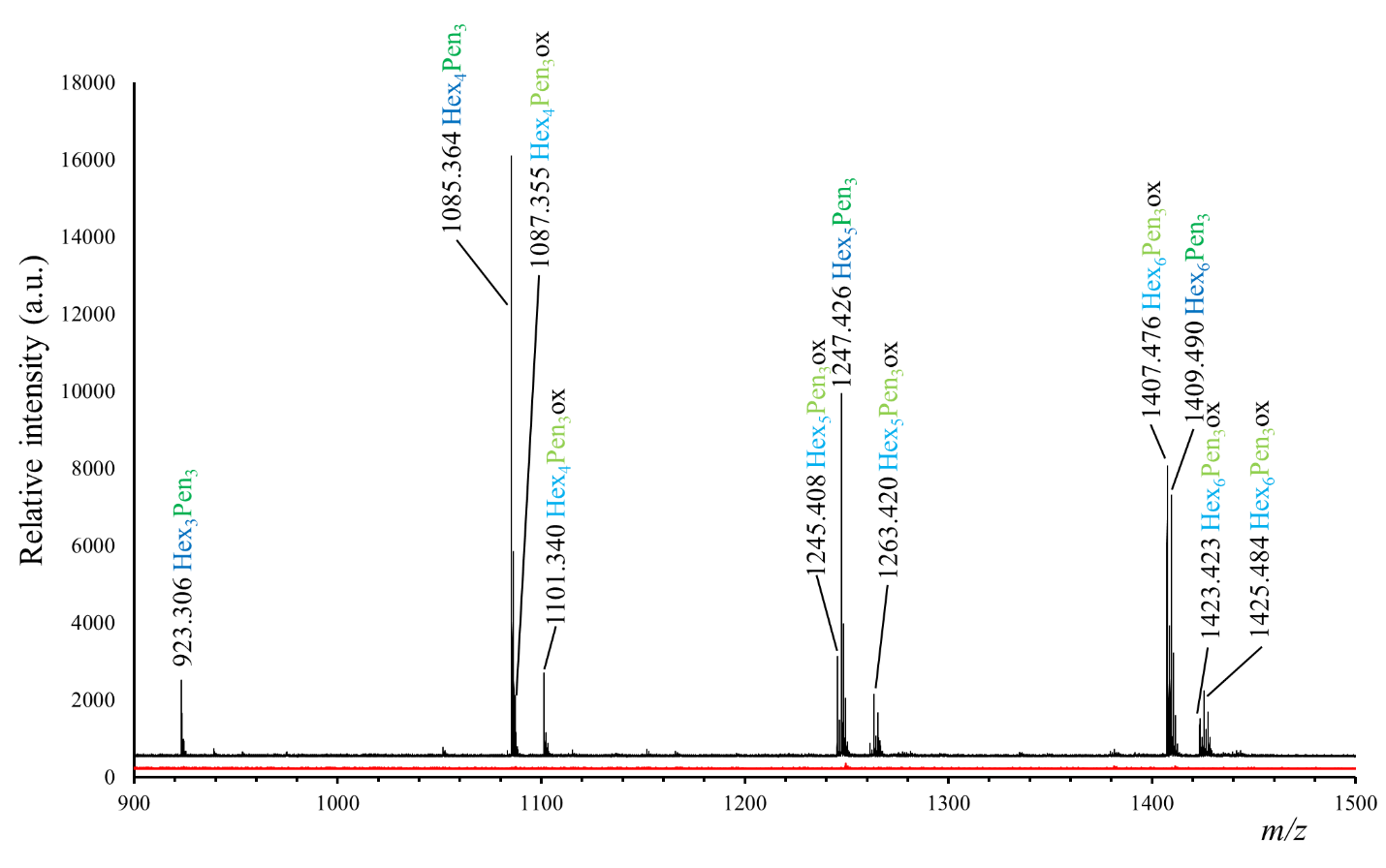
**Figure S2. MALDI-TOF analysis of products generated in reactions of *Sc*LPMO9A with XG14 in the presence of AscA.** The spectrum shows the samples analyzed with HPAEC-PAD in **Figure 7B** in the main text. The red spectrum shows the corresponding reaction without AscA. The labeled products are the sodium adducts of native and oxidized species, the formation of which is reductant-dependent. The oxidized keto (-2 *m/z* from the native) and geminal diol (+16 *m/z* from the native) forms of Hex_5_Pen_3_ (XXLG) and Hex_6_Pen_3_ (XLLG) were detected, but only the geminal diol form was detected for oxidized Hex_4_Pen_3_ (XXXG); the signal possibly reflecting this hydrated oxidized product (*m/z* 1101.3) may also represent a potassium adduct of non-oxidized XXXG.


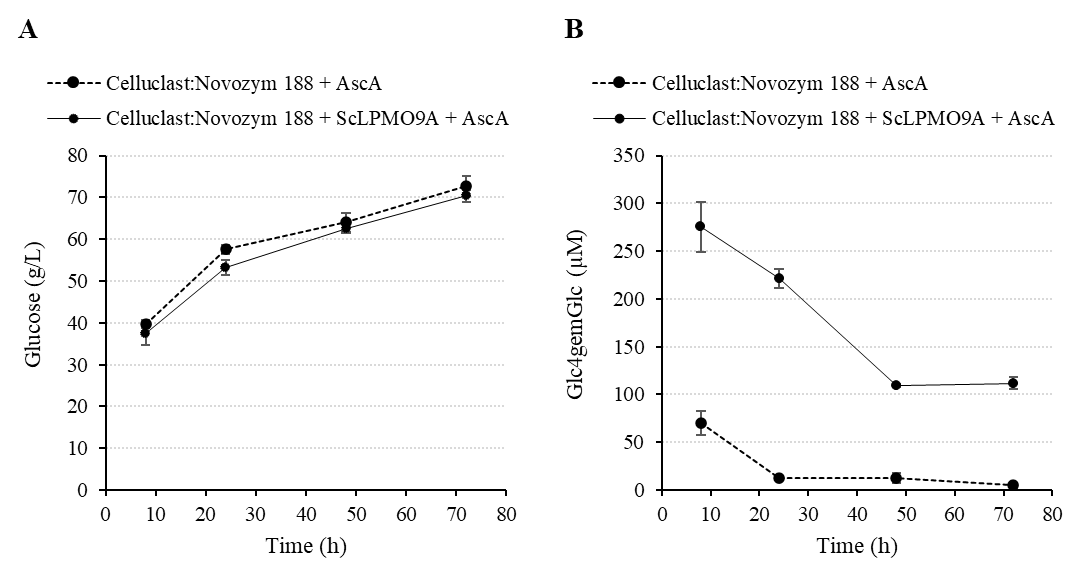


**Figure S3. Degradation of sulfite-pulped spruce by an LPMO-poor cellulase blend with or without added *Sc*LPMO9A.** Panel A shows glucose yield, and Panel B shows production of Glc4gemGlc. The substrate (10% w/w dry matter) was incubated with a Celluclast:Novozym 188 blend in 50 mM sodium acetate pH 5.0. In reactions with LPMO, 10% of this blend was replaced with *Sc*LPMO9A (on a protein basis). Reactions were initiated by adding 1 mM AscA, and incubated at 50°C with orbital shaking at 200 rpm. Error bars indicate standard deviations between triplicates.

**
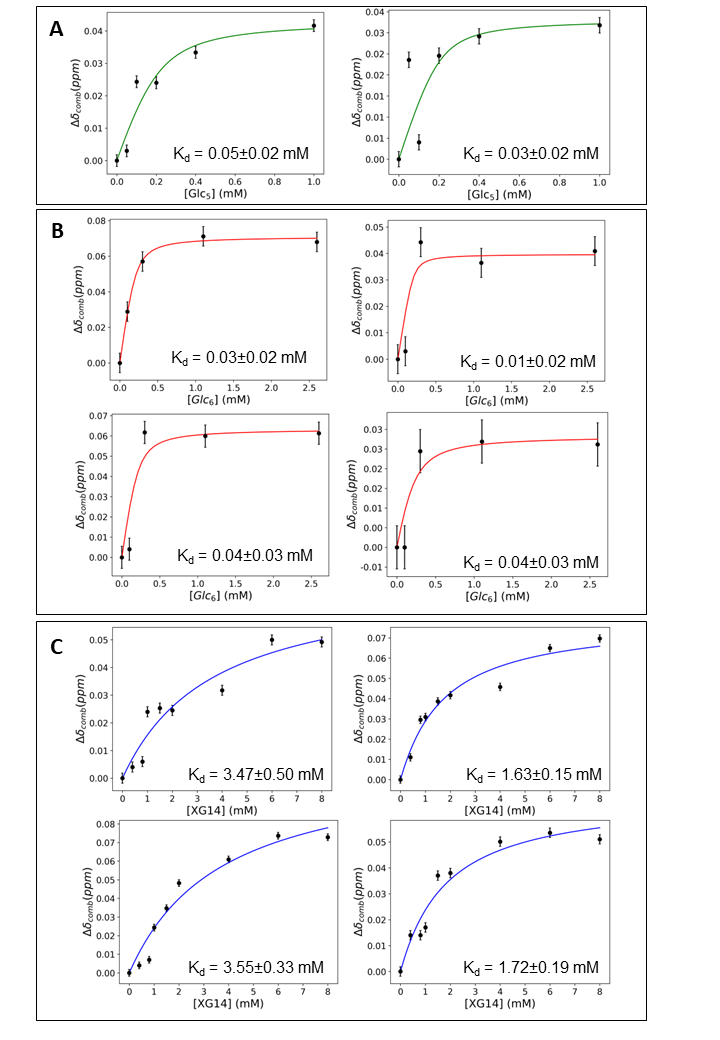
**

**Figure S4. Titration experiments between *Sc*LPMO9A and cellopentaose (Glc_5_), cellohexaose (Glc_6_), and xyloglucan tetradecamer (XG14).** The dissociation constant (K_d_) was calculated by plotting the combined chemical shift perturbation (Δδ_comb_ in ppm) against the substrate concentration (mM). Panel A: estimation of the K_d_ when *Sc*LPMO9A was titrated with 0.05, 0.1, 0.2, 0.4, and 1 mM cellopentaose. Panel B: estimation of the K_d_ when *Sc*LPMO9A was titrated with 0.1, 0.3, 0.5, 1.1, and 2.5 mM cellohexaose. Panel C: estimation of the K_d_ when *Sc*LPMO9A was titrated with 0.4, 0.8, 1.0, 1.5, 2.0, 4.0, 6.0, and 8 mM XG14.


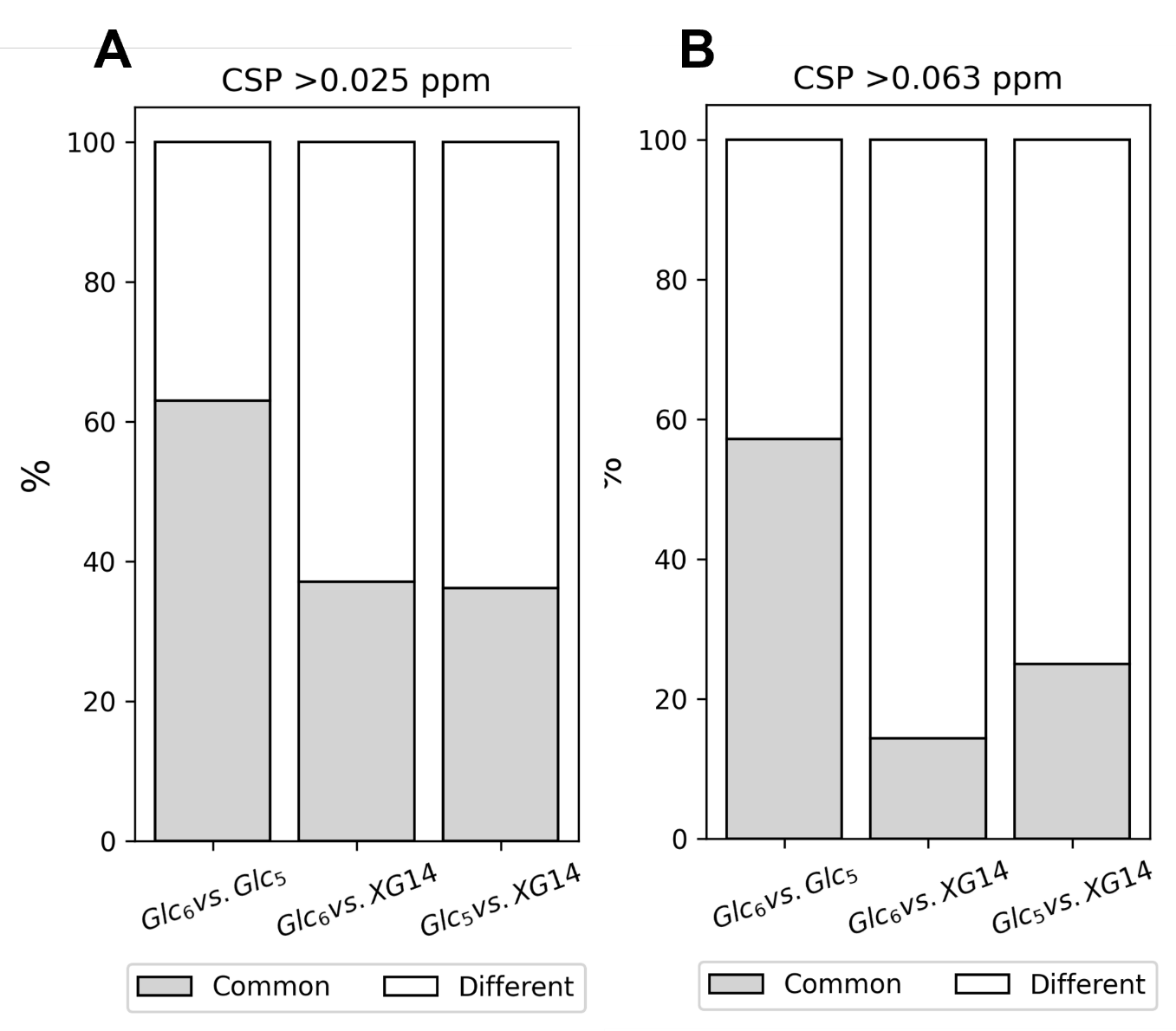


**Figure S5. Comparison of residues in *Sc*LPMO9A whose ^1^H-^15^N signal is affected by interaction with cellopentaose, cellohexaose, and XG14.** Panel A: Stacked bar plots showing the percentage of common and different residues with ^1^H-^15^N signals showing a CSP ≥ 0.025 ppm when titrated with 2.5 mM cellohexaose, 1.0 mM cellopentaose, or 8.0 mM XG14. Panel B: Stacked bar plots showing the percentage of common and different residues with ^1^H-^15^N signals showing a CSP ≥ 0.063 ppm when titrated with 2.5 mM cellohexaose, 1.0 mM cellopentaose, or 8.0 mM XG14. In the two compared titration experiments, 100% is given as the combined number of unique residues showing a CSP > 0.025 ppm and > 0.063 ppm, respectively.
